# The peroxisome proliferator-activated receptor agonist pioglitazone and 5-lipoxygenase inhibitor zileuton have no effect on lung inflammation in healthy volunteers by positron emission tomography in a single-blind placebo-controlled cohort study

**DOI:** 10.1371/journal.pone.0191783

**Published:** 2018-02-07

**Authors:** Delphine L. Chen, Howard J. Huang, Derek E. Byers, Adrian Shifren, Bryan Belikoff, Jacquelyn T. Engle, Elizabeth Arentson, Debra Kemp, Sharon Phillips, David E. Scherrer, Hideji Fujiwara, Katherine J. Spayd, Frank J. Brooks, Richard A. Pierce, Mario Castro, Warren Isakow

**Affiliations:** 1 Mallinckrodt Institute of Radiology, Division of Radiological Sciences, Washington University School of Medicine, St. Louis, MO, United States of America; 2 Department of Internal Medicine, Division of Pulmonary and Critical Care Medicine, Washington University School of Medicine, St. Louis, MO, United States of America; 3 Annette C. and Harold C. Simmons Transplant Institute, Baylor University Medical Center at Dallas, Dallas, TX, United States of America; 4 Center for Clinical Studies, Washington University School of Medicine, St. Louis, MO, United States of America; 5 Department of Internal Medicine, Cardiovascular Division, Washington University School of Medicine, St. Louis, MO, United States of America; Public Library of Science, UNITED KINGDOM

## Abstract

**Background:**

Anti-inflammatory drug development efforts for lung disease have been hampered in part by the lack of noninvasive inflammation biomarkers and the limited ability of animal models to predict efficacy in humans. We used ^18^F-fluorodeoxyglucose (^18^F-FDG) positron emission tomography (PET) in a human model of lung inflammation to assess whether pioglitazone, a peroxisome proliferator-activated receptor-γ (PPAR-γ) agonist, and zileuton, a 5-lipoxygenase inhibitor, reduce lung inflammation.

**Methods:**

For this single center, single-blind, placebo-controlled cohort study, we enrolled healthy volunteers sequentially into the following treatment cohorts (N = 6 per cohort): pioglitazone plus placebo, zileuton plus placebo, or dual placebo prior to bronchoscopic endotoxin instillation. ^18^F-FDG uptake pre- and post-endotoxin was quantified as the Patlak graphical analysis-determined *K*_i_ (primary outcome measure). Secondary outcome measures included the mean standard uptake value (SUV_mean_), post-endotoxin bronchoalveolar lavage (BAL) cell counts and differentials and blood adiponectin and urinary leukotriene E_4_ (LTE_4_) levels, determined by enzyme-linked immunosorbent assay, to verify treatment compliance. One- or two-way analysis of variance assessed for differences among cohorts in the outcome measures (expressed as mean ± standard deviation).

**Results:**

Ten females and eight males (29±6 years of age) completed all study procedures except for one volunteer who did not complete the post-endotoxin BAL. *K*_i_ and SUV_mean_ increased in all cohorts after endotoxin instillation (*K*_i_ increased by 0.0021±0.0019, 0.0023±0.0017, and 0.0024±0.0020 and SUV_mean_ by 0.47±0.14, 0.55±0.15, and 0.54±0.38 in placebo, pioglitazone, and zileuton cohorts, respectively, p<0.001) with no differences among treatment cohorts (p = 0.933). Adiponectin levels increased as expected with pioglitazone treatment but not urinary LTE_4_ levels as expected with zileuton treatment. BAL cell counts (p = 0.442) and neutrophil percentage (p = 0.773) were similar among the treatment cohorts.

**Conclusions:**

Endotoxin-induced lung inflammation in humans is not responsive to pioglitazone or zileuton, highlighting the challenge in translating anti-inflammatory drug efficacy results from murine models to humans.

**Trial registration:**

ClinicalTrials.gov NCT01174056.

## Introduction

Lung diseases contribute significantly to overall morbidity and mortality. Chronic lower respiratory diseases, such as chronic obstructive pulmonary disease (COPD) and asthma, are the third leading cause of death in the US [1]. In COPD and asthma, as well as acute respiratory distress syndrome (ARDS), increased neutrophils are seen in the lungs, and increasing numbers of lung neutrophils correlate with disease severity [2–11]. Inhaled and systemic corticosteroid therapy has therefore been a mainstay for treating these diseases; however, certain phenotypes of these disease are insensitive to steroid therapy [12]. Efforts to identify new anti-inflammatory treatments to overcome such treatment resistance or to reduce the functional impact of lung disease, however, have met with limited success [13–16].

Two factors contribute in part to the limited successes of lung anti-inflammatory drug development. One is the inability of animal models to predict whether pulmonary anti-inflammatory drugs will be effective in humans has contributed in part to the difficulties of drug development in this area [17, 18]. Therefore, human models would potentially be of greater value for studying inflammatory pathways and may better predict the efficacy of pulmonary anti-inflammatory drugs. One such model of experimentally induced lung inflammation using endobronchially instilled endotoxin in healthy volunteers was developed for this purpose [19]. In this model, the endotoxin leads to a self-limited, neutrophilic inflammatory response that exhibits proteomic responses similar to those seen in ARDS patients [20]. Therefore, this model could be used to assess the effects of anti-inflammatory drugs in humans.

Another is the lack of noninvasive, quantitative biomarkers that accurately reflect the burden of inflammation in the lungs further hampers anti-inflammatory drug development efforts [13]. PET imaging with ^18^F-fluorodeoxyglucose (^18^F-FDG) has been used to image the lungs’ inflammatory burden in ARDS and COPD patients [21–24]. PET imaging of ^18^F-FDG uptake can also detect the mild lung inflammation induced by endobronchial-instilled endotoxin in healthy volunteers [25] and has been used to demonstrate the anti-inflammatory effect of the cholesterol-lowering drug lovastatin in this model [26]. Therefore, using this human model with ^18^F-FDG PET imaging could be a useful way to screen drugs for anti-inflammatory efficacy prior to evaluation in larger and more complicated patient clinical trials.

Multiple studies suggest that the peroxisome proliferator-activated receptor-γ (PPAR-γ) agonist pioglitazone reduces inflammation in animal models of lung injury, at least in part by blocking the production of the neutrophil chemoattractant and activator IL-8 [27–31]. Treatment with the 5-lipoxygenase inhibitor zileuton has also been shown to reduce inflammation by blocking the production of the neutrophil chemoattractant leukotriene B4 (LTB_4_) [32, 33]. Given that drug pharmacokinetics are known to vary among animals and humans [34, 35], we designed this study to determine whether these anti-inflammatory effects observed in animal models would also be observed in humans. This study tested whether pioglitazone or zileuton as single agents could reduce endotoxin-induced lung inflammation in healthy volunteers. ^18^F-FDG uptake, quantified as the *K*_i_ determined by Patlak graphical analysis, was the primary outcome measure. BAL cell counts and peripheral blood clinical parameters were secondary outcome measures, and mean standard uptake value (SUV_mean_) for quantifying ^18^F-FDG uptake and BAL fluid assays were exploratory outcome measures.

## Materials and methods

### Ethics, consent, and permissions

This study was approved by the Washington University Institutional Review Board (protocol #201101731) and conducted under Investigational New Drug application #100042 for endotoxin. All volunteers gave written informed consent to participate. This trial was registered on clinicaltrials.gov (#NCT01174056) and conducted according to the principles expressed in the Declaration of Helsinki.

### Study design, participant and procedure flow

**Fig 1:**
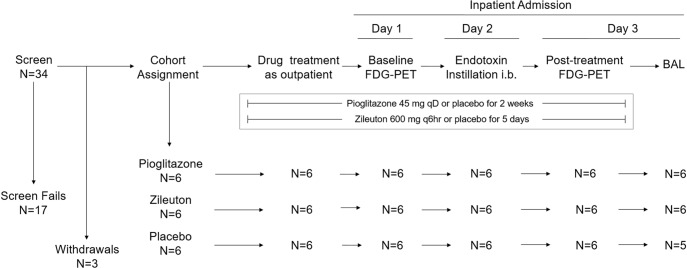
Study design, participant and procedure flow. Reasons for screen failures were: Elevated alanine aminotransferase level (N = 5); % predicted forced expiratory volume in one second (FEV1) and/or forced vital capacity (FVC) < 90% (N = 6); minimal airway obstruction on pulmonary function testing (N = 1); abnormal chest x-ray (N = 1); abnormal electrocardiogram (N = 1); history of childhood asthma (N = 1); smoking within 1 year of enrollment (N = 1); body mass index outside range (N = 1). Withdrawals were volunteers who signed the consent after completing the screening procedures and were given the blinded study drugs but withdrew from the study prior to initiating the imaging and bronchoscopy procedures and were lost to follow up. FDG-PET = positron emission tomography imaging with ^18^F-fluorodeoxyglucose. i.b. = intrabronchial. qD = once per day. Q6hr = once every six hours.

This study was approved by the Washington University Institutional Review Board (protocol #201101731) and conducted under Investigational New Drug application #100042 for endotoxin. All volunteers provided written informed consent to participate. We conducted a single-center, single-blind, placebo-controlled cohort study from February 2012 to March 2014 with volunteers enrolled sequentially into the following treatment groups, in order: 1) pioglitazone plus matching placebo for zileuton (pioglitazone cohort), 2) matching placebo for pioglitazone plus zileuton (zileuton cohort), 3) placebo plus placebo (placebo cohort). Eligible participants had no history of cardiopulmonary disease and normal results on screening spirometry, chest radiograph, electrocardiogram, and bloodwork. Fig 1 shows the study procedure outline. FDG-PET imaging was performed the day before and at approximately 24 hours after endotoxin instillation. See S1 File for additional methods.

This study was originally designed with a fourth cohort to test combined treatment with pioglitazone and zileuton based on data showing that 5-lipoxygenase inhibition blocked rosiglitazone’s anti-inflammatory effect in a rodent stroke model [36]. To reduce unnecessary study drug exposure in healthy volunteers in the event that pioglitazone had no anti-inflammatory effect, we conducted a planned interim analysis comparing the *K*_i_ values in the pioglitazone cohort to previously published post-endotoxin control values [25, 26], which showed no difference. Therefore, volunteers were enrolled only into the single-agent pioglitazone and zileuton treatment cohorts as well as the placebo cohort.

### Treatments

Endotoxin (*Escherichia coli* O113:H10K) was obtained from the National Institutes of Health (NIH) Clinical Center and instilled bronchoscopically (4 ng/kg) in the right middle lobe as previously described [26]. Pioglitazone (Takeda Pharmaceuticals, 45 mg/day orally for two weeks) and zileuton (Cornerstone Pharmaceuticals, 600 mg orally every six hours for five days) were given prior to endotoxin instillation, according to the schedule shown in Fig 1. Both drugs were purchased commercially and over-encapsulated to match the placebo for blinding purposes. Volunteers were enrolled into each treatment cohort sequentially with N = 6 in each group.

### FDG-PET image acquisition and analysis

Sixty minutes of dynamic PET images were obtained on a Siemens Biograph 40 PET/CT scanner after intravenously injecting 370 ± 18 MBq (10.0 ± 0.5 mCi) of ^18^F-FDG. Venous blood samples were obtained throughout the scan as previously described [25, 26]. A low-dose computed tomography (CT) scan was obtained for attenuation correction of the PET images. All scans were analyzed using Integrated Research Workflow 4.0 (Siemens) as previously described [25, 26, 37]. Briefly, the baseline and post-endotoxin PET and CT scans were coregistered. Volumes of interest (VOIs) were then placed on the CT images in areas of post-procedure airspace inflammation and transferred to the PET images to extract the time-activity curves. The Patlak graphical analysis was used to calculate the influx constant *K*_i_ [38, 39]. The SUV_mean_ at 60 min after tracer injection was quantified for ^18^F-FDG uptake from the same VOIs used for the Patlak analysis.

### BAL procedures, assays and analysis

BAL was performed and the fluid processed as previously described [37]. All three retrieved aliquots were pooled into a single sample. BAL cell counts and differentials were determined as previously described [25, 37]. BAL fluid filtered through gauze was processed and stored at -80° C until ready for analysis. Given that the interim analysis was negative for the primary outcome measure, assays for 5- and 15-hydroxyeicosatetraenoic acid (5-HETE and 15-HETE, respectively), lipoxin A_4_ (LXA_4_), LTB_4_, and LTE_4_ were performed as exploratory analyses on a subset of BAL fluid samples.

### Blood and urine assays

Serum and urine obtained at the screening visit and on the day before endotoxin instillation were kept frozen at -80^o^ C. Serum adiponectin levels were measured using an enzyme-linked immunosorbent assay (ELISA) kit (Millipore, catalog #EZHADP-61K) according to the manufacturer’s instructions. Levels of urinary LTE_4_ were measured by ELISA (Cayman Chemical, catalog #520411) as previously described [40] and normalized for creatinine, measured by mass spectrometry as described in the S1 File. Toll-like receptor 4 (TLR4) single nucleotide polymorphisms (SNPs) Asp299Gly (rs4986790) and Thr399Ile (rs4986791), associated with decreased endotoxin responsiveness, were tested in all volunteers [41]. DNA was extracted from whole blood using the PureGene protocol (Qiagen) according to the manufacturer’s instructions and sent for genotyping by DNA Genotek, Inc. (Kanata, Ontario).

### Statistical analysis

A sample size of six per group was chosen based on data from prior studies [25, 26]. A one-way analysis of variance (ANOVA) was utilized to compare baseline characteristics among all treatment cohorts (SigmaPlot 12.5, Systat Software, Inc.). Two-way repeated measures ANOVA with endotoxin status (pre- or post-instillation) and treatment cohort as covariates and Tukey’s method for post-hoc analysis (when applicable) was used to assess for endotoxin-induced changes in clinical parameters (including vital signs, cellular differentials, and pulmonary function tests), *K*_i_ and SUV_mean_ from the PET data, and adiponectin and uLTE_4_ levels. A few *K*_i_ values were slightly negative; these were set to zero for the analysis. Bonferroni correction for multiple comparisons was applied to the statistical results for the clinical parameters. Differences in the BAL cell counts among the cohorts was determined by one-way ANOVA.

## Results

### Participant flow and clinical characteristics

We enrolled 38 volunteers, with 17 failing screen procedures and three withdrawing consent after screen, leaving 18 total volunteers who completed all imaging procedures. The endotoxin was instilled in the lateral segment of the right middle lobe in all volunteers except for one volunteer in whom it was instilled in the medial segment. One volunteer in the placebo cohort experienced a prolonged recovery period from the anesthesia for the endotoxin instillation bronchoscopy; therefore, the BAL bronchoscopy procedure for this volunteer was canceled, leaving 17 volunteers who completed all study procedures (both imaging and bronchoscopy). The participant flow is summarized in Fig 1. All volunteers reported at least one of a number of expected mildly severe symptoms, such as joint aches, sore throat, and cough, after the bronchoscopy for endotoxin instillation. No unexpected or serious adverse events related to the study occurred during the course of the trial. Detailed adverse event reporting is listed in the S2 File.

No significant differences among the cohorts were noted in the baseline clinical characteristics of the volunteers with evaluable image data (Table 1). The frequency of symptoms and changes in clinical parameters after endotoxin did not differ among the cohorts except for the percentage of blood neutrophils, which increased significantly only in the placebo group (Table 2). Significantly increased leukocytosis, peripheral blood neutrophilia, and C-reactive protein, but not erythrocyte sedimentation rate, were observed after endotoxin in all cohorts. No significant changes in lung function measures were noted.

**Table 1:** Baseline clinical characteristics.

Parameter*	Placebo (N = 6)	Pioglitazone (N = 6)	Zileuton (N = 6)	P-value
Age (years)	29 ± 3	31 ± 6	31 ± 8	0.891
Gender	3F/3M	3F/3M	4F/2M	N/D
Race/Ethnicity	4 African-American; 1 African-American/Latino; 1 Caucasian	2 African-American; 4 Caucasian	2 African- American; 4 Caucasian	N/D
Vital Signs				
Temperature, °C	36.8 ± 0.4	36.8 ± 0.3	36.3 ± 0.4	0.031
Heart rate, beats/min	68 ± 6	73 ± 12	74 ± 9	0.557
Blood pressure, mm HG, systolic	117 ± 6	121 ± 16	119 ± 10	0.841
Blood pressure, mm HG, diastolic	74 ± 6	77 ± 8	74 ± 6	0.713
Mean arterial pressure, mm Hg	88 ± 5	92 ± 11	89 ± 7	0.753
S_a_O_2_, % on room air	99 ± 1	98 ± 1	99 ± 1	0.250
Respiratory rate, breaths/min	18 ± 2	17 ± 1	17 ± 2	0.853
Pulmonary function tests				
FEV1, L	3.3 ± 1.5	4.1 ± 0.7	3.7 ± 0.7	0.853
% predicted FEV_1_	98 ± 5	105 ± 10	103 ± 9	0.294
FVC, L	3.8 ± 0.6	5.1 ± 1.1	4.7 ± 0.7	0.059
% predicted FVC	97 ± 6	108 ± 10	110 ± 14	0.081
Complete blood counts				
White blood cells, 10^3^/ml	5.4 ± 2.0	5.8 ± 1.4	6.6 ± 1.0	0.400
% neutrophils	52 ± 8	61± 10	63 ± 6	0.079
Hemoglobin, g/dl	13 ± 1	13 ± 1	13 ± 2	0.954
Hematocrit, %	39 ± 3	38 ± 4	39 ± 5	0.903
Platelets, 1000/mm^3^	246 ± 40	237 ± 55	226 ± 61	0.819
Erythrocyte sedimentation rate (ESR), mm/hr	6.2 ± 2.5	8.0 ± 1.9	7.0 ± 5.5	0.689
C-reactive protein (CRP), mg/L	0.9 ± 0.9	0.5 ± 0.7	0.6 ± 0.6	0.429

*No significant differences were found. p < 0.003 required for statistical significance with Bonferroni correction for multiple comparisons. Statistical testing was not performed for gender or racial/ethnicity distributions (N/D).

Data shown as mean ± standard deviation.

FEV1: Forced expiratory volume in 1 second

FVC: Forced vital capacity

**Table 2:** Effect of endotoxin on clinical characteristics.

Change after endotoxin				
Parameter	Placebo (N = 6)	Pioglitazone (N = 6)	Zileuton (N = 6)	P-value
Vital Signs				
Temperature, °C	0.5 ± 0.3	0.8 ± 0.6	0.7 ± 0.6	<0.001*
Heart rate, beats/min				
Compared to highest	21 ± 5	23 ± 11	22 ± 6	<0.001*
Compared to lowest	−7 ± 5	−10 ± 9	−6 ± 4	<0.001*
Blood pressure, mm HG, systolic	−19 ± 10	−19 ± 5	−17 ± 9	<0.001*
Blood pressure, mm HG, diastolic	−18 ± 7	−23 ± 7	−20 ± 6	<0.001*
Mean arterial pressure, mm Hg	−14 ± 6	−20 ± 4	−18 ± 7	<0.001*
S_a_O_2_, % on room air	−2.7 ± 1.2	−2.8 ± 1.5	1.7 ± 1.2	<0.001*
Respiratory rate, breaths/min	3 ± 2	2 ± 3	4 ± 2	<0.001*
Pulmonary function tests				
FEV_1_, L	−0.2 ± 0.3	−0.2 ± 0.1	0.0 ± 0.1	0.006
% predicted FEV_1_	−5.5 ± 7.5	−5.2 ± 4.4	-0.2 ± 2.8	0.011
FVC, L	−0.2 ± 0.2	−0.1 ± 0.0	0.0 ± 0.1	0.008
% predicted FVC	−4.8 ± 5.8	−2.6 ± 1.6	0.1 ± 3.0	0.021
Complete blood counts				
White blood cells, 10^3^/ml	3.9 ± 1.2	1.7 ± 1.1	3.2 ± 2.4	<0.001*
% neutrophils	17.9 ± 8.6**	3.4 ± 6.4	4.4 ± 7.3	<0.001**
Hemoglobin, g/dl	0.1 ± 0.9	−0.6 ± 0.5	−0.1 ± 0.5	0.257
Hematocrit, %	0.5 ± 2.5	−1.7 ± 1.7	−0.2 ± 2.0	0.376
Platelets, 1000/mm^3^	−12 ± 24	−31 ± 19	−22 ± 22	<0.001*
Erythrocyte sedimentation rate (ESR), mm/hr	−1.2 ± 1.9	−0.7 ± 1.0	−0.5 ± 1.1	0.033
C-reactive protein (CRP), mg/L	10.1 ± 7.3	6.9 ± 5.6	6.3 ± 6.5	<0.001*

*p < 0.003 required for statistical significance with Bonferroni correction for multiple comparisons. P values shown for comparisons of values before and after endotoxin across all groups in the repeated measures analysis of variance. No interaction was found between drug treatment and endotoxin effect on any of the parameters except for the % neutrophils in the peripheral blood.

**p < 0.001 when comparing the post-endotoxin value to the pre-endotoxin value within the placebo cohort. No significant difference was found in pre- and post-endotoxin % neutrophil values in the other two treatment groups (p = 0.29 and 0.17 for the pioglitazone and zileuton treatment cohorts, respectively).

Data shown as mean ± standard deviation.

### Pioglitazone and zileuton effect on ^18^F-FDG uptake

**Fig 2:**
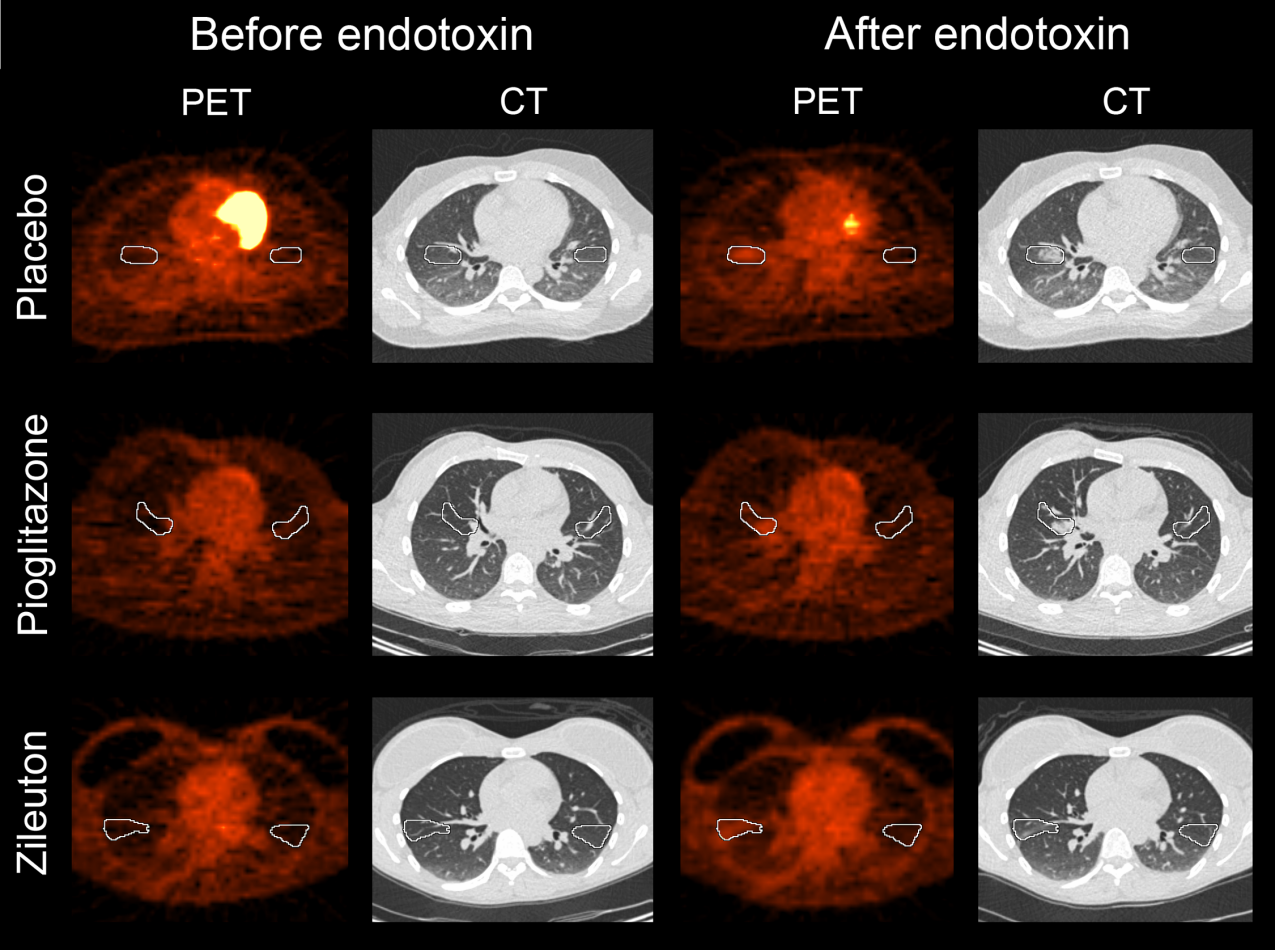
Positron emission tomography (PET) and computed tomography (CT) images from a representative volunteer in each treatment cohort. White outlines show the volumes of interest (VOIs) used to determine the time-activity curves for the Patlak graphical analysis and standard uptake values. VOIs were sometimes smaller in volume on the left due to the heart.

**Fig 3:**
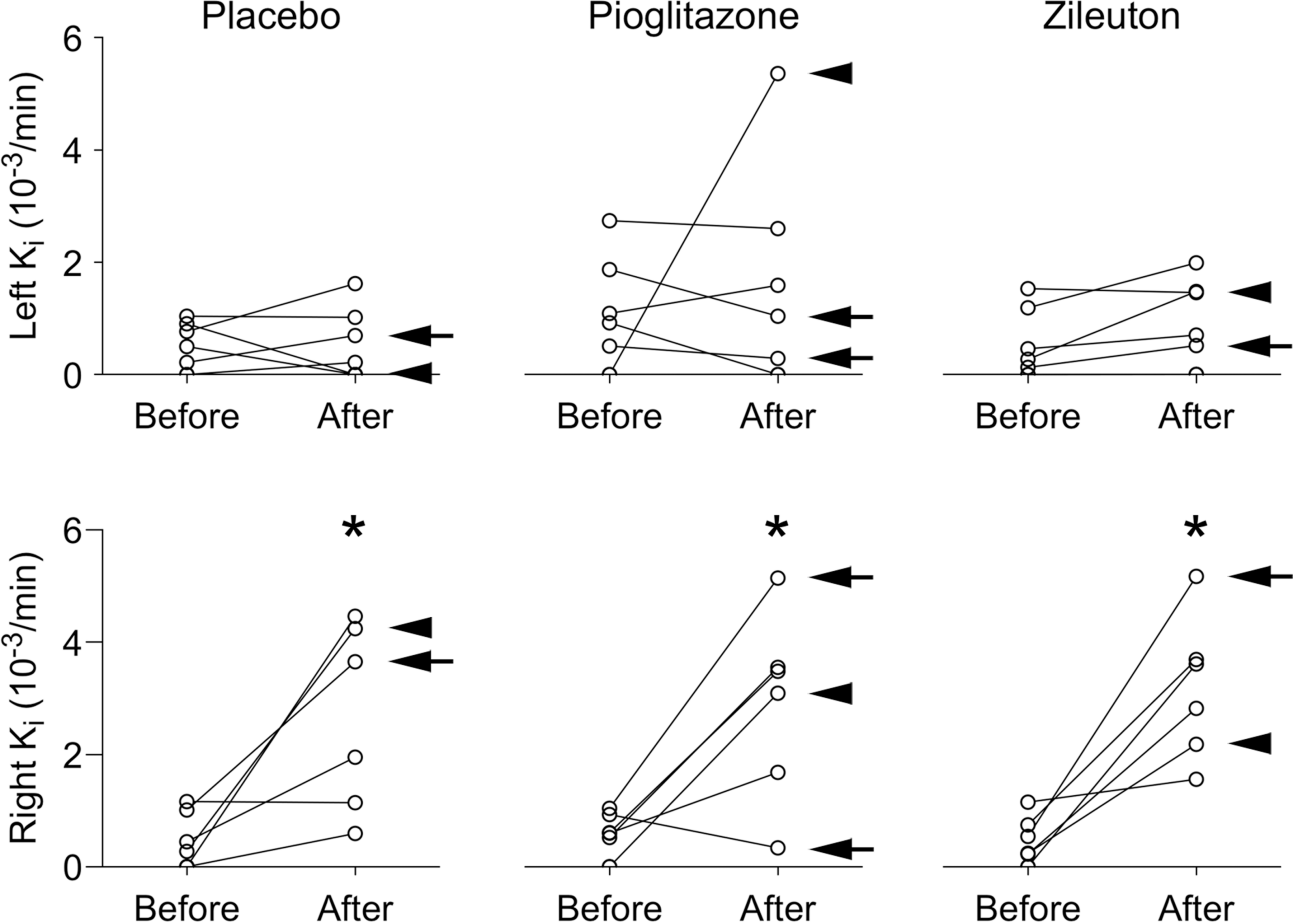
Patlak graphical analysis results from^18^F-fluorodeoxyglucose (^18^F-FDG) PET images in the right and left lungs before and after endotoxin instillation. Arrows indicate presence of both Asp299Gly and Thr399Ile single nucleotide polymorphisms (SNPs), arrowheads only the Asp299Gly SNP. The arrowhead for the left lung data of the zileuton treatment cohort points to the post-endotoxin data point that decreased slightly after endotoxin. *K*_i_ = influx constant describing rate of ^18^F-FDG uptake into the lung region of interest, determined by Patlak graphical analysis. * = p < 0.05 when comparing post-endotoxin (After) to pre-endotoxin (Before) value.

**Fig 4:**
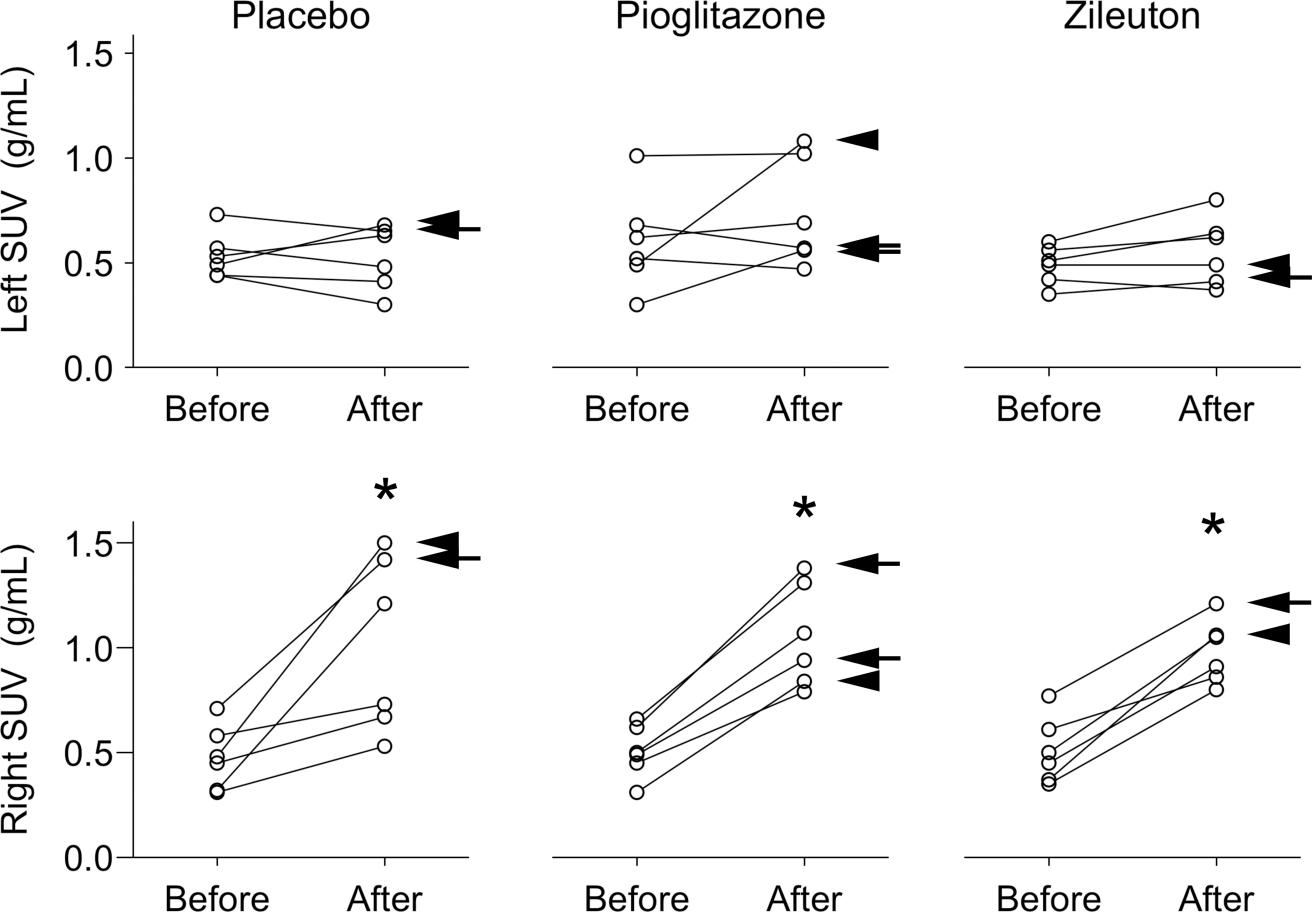
Mean standard uptake value (SUV) results from^18^F-fluorodeoxyglucose PET images in the right and left lungs for each treatment cohort. Arrows indicate presence of both Asp299Gly and Thr399Ile single nucleotide polymorphisms (SNPs), arrowheads only the Asp299Gly SNP. The arrow and arrowhead for the left lung of the placebo cohort point to the top two post-endotoxin data points. * = p < 0.05 when comparing post-endotoxin (After) to pre-endotoxin (Before) value.

Representative PET/CT images are shown in Fig 2. Post-procedure airspace inflammation were noted on the CT images in all volunteers after endotoxin instillation. The average CT volumes and numbers of PET voxels within the VOIs was similar across cohorts and is summarized in the S1 Table. The interim analysis comparing pre- and post-endotoxin *K*_i_ values in the right middle lobe in the pioglitazone group (pre-endotoxin 0.00062±0.00037, post-endotoxin 0.0029±0.0017) compared to previously reported *K*_i_ values in healthy volunteers receiving endotoxin and either no drug treatment or placebo treatment from two prior studies [25, 26] (pre-endotoxin 0.00046±0.00044, post-endotoxin 0.0026±0.0010, in aggregate, N = 12) showed no differences among groups (p = 0.072 for two-way RM ANOVA). Based on the interim analysis, we concluded that pioglitazone had no anti-inflammatory effect. Increases in right middle lobe post-endotoxin *K*_i_ (placebo cohort: pre-endotoxin 0.00048±0.00050, post-endotoxin 0.0026±0.0018; zileuton cohort: pre-endotoxin 0.00048±0.00042, post-endotoxin 0.0029±0.0017) and SUV_mean_ (placebo cohort: pre-endotoxin 0.48±0.15, post-endotoxin 1.01±0.42; pioglitazone cohort: pre-endotoxin 0.51±0.13, post-endotoxin 1.06±0.25; zileuton cohort: pre-endotoxin 0.51±0.16, post-endotoxin 0.98±0.15) were similar in all volunteers (p < 0.001), regardless of the drug treatment received or presence of TLR4 SNP (Fig 3 and Fig 4). *K*_i_ values were more variable than seen on prior studies [25, 26].

### BAL cell count changes in response to drug treatments

The percentage of neutrophils in the BAL cells (Table 3) increased in all treatment cohorts after endotoxin instillation, as has been observed previously [25]. The lower mean total cell and neutrophil counts and much larger standard deviation in the total BAL cell counts in the pioglitazone cohort was due to a single low outlier value that was included in the analysis. BAL fluid analyses demonstrated no differences in 5-HETE, 15-HETE, LXA_4_, LTB_4_, and LTE_4_ levels among groups. This may have been due to the fact that the BAL was performed at approximately 29 hours after endotoxin instillation, long after the peak cytokine increase normally seen at six hours post-endotoxin in this model [19]. These results are presented in more detail in the S3 File.

**Table 3:** BAL cell counts and differentials.

BAL Measures	Placebo (N = 5)	Pioglitazone (N = 6)	Zileuton (N = 6)
Total cell count, cells/mm^3^	7060 ± 3904	4879 ± 5063	8410 ± 4862
% neutrophils	48 ± 23	50 ± 21	58 ± 12
% monocytes	50 ± 20	49 ± 21	39 ± 12
% others	2 ± 3	0.95 ± 0.74	3 ± 3
Neutrophil concentration, cells/mm^3^	4099 ± 2492	2787 ± 2841	5179 ± 3629

Values given as mean ± standard deviation

BAL: Bronchoalveolar lavage

### Blood and urine assays confirm compliance with prescribed drug treatments

**Fig 5:**
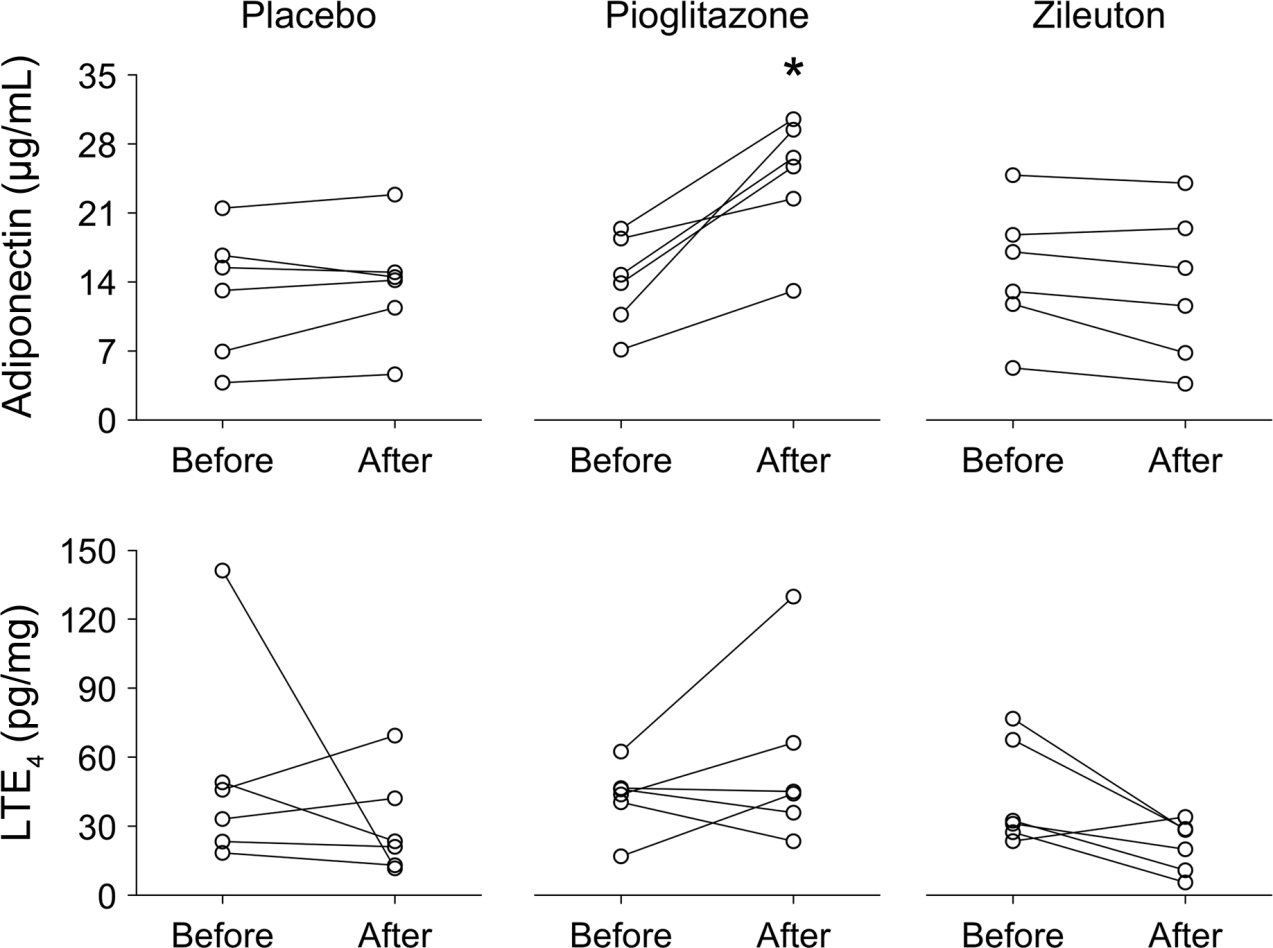
Serum adiponectin and urinary leukotriene E4 (LTE_4_) levels by treatment cohort. * = p < 0.05 when comparing post-endotoxin (After) to pre-endotoxin (Before) values. Serum adiponectin levels are expressed as mg/mL serum. Urinary LTE_4_ levels are expressed as pg LTE_4_/mg creatinine (pg/mg).

Plasma adiponectin significantly increased only in the pioglitazone cohort. No significant differences in urinary LTE_4_ levels were noted, though the post-treatment values dropped more consistently in the zileuton cohort (Fig 5).

## Discussion

In this study, we have shown that pioglitazone and zileuton have no effect on endotoxin-induced lung inflammation in healthy volunteers using ^18^F-FDG PET/CT imaging. We observed increased ^18^F-FDG uptake in all treatment cohorts that was similar that observed in prior studies using this same model [25, 26]. The increase in BAL cell counts, and the variability associated with this measurement, was also similar to previous studies [19, 25, 26]. These results contrast with data in animal models demonstrating their efficacy as pulmonary anti-inflammatory treatments [27–33]. The expected changes in plasma adiponectin and urinary LTE_4_ levels suggested that noncompliance with the drug treatment regimen was unlikely to explain this result. Neither drug significantly reduced BAL cell counts or blood C-reactive protein levels after endotoxin instillation as well, further confirming that these drugs had no anti-inflammatory effect in this model.

Our results continue to support the utility of ^18^F-FDG PET imaging as a noninvasive biomarker of lung inflammation and suggest that the SUV_mean_ determined from PET/CT images may be sufficient for detecting the low-level lung inflammation induced in this human model. We have used the *K*_i_ in previous studies performed on a dedicated PET scanner (Siemens ECAT EXACT HR+) to quantify the rate of ^18^F-FDG uptake in endotoxin-induced inflammation in healthy volunteers [25, 26]. We showed previously that the SUV_mean_ is less accurate for quantifying low levels of inflammation in a dog model of ARDS [42]. However, in this study, we observed a consistent increase in SUV_mean_ after endotoxin instillation, similar to our previously published data with *K*_i_ [25, 26]. The SUV_mean_ was also less variable than the *K*_i_ in this study. In our previous studies, the attenuation correction transmission scan on the dedicated PET scanner was acquired over several minutes, thus averaging multiple respiratory cycles in the same manner as the PET emission data. In this study, we observed irregularities in a few of the lung time-activity curves used for the Patlak analysis, thus causing these *K*_i_ values to be slightly negative. These irregularities were most likely due to PET-CT misregistration errors from respiratory motion during the one-hour acquisition, which can lead to small attenuation correction errors [43]. The SUV_mean_ observed in the smaller VOIs, defined by the degree of airspace inflammation seen on the CT images, may have been lower because of such motion as well as partial-volume averaging error; however, we were still able to detect increased uptake in these VOIs. The PET and CT images appeared well-matched at the end of the one-hour acquisition on all subjects, which likely helped explain the more consistent increase seen in the SUV_mean_. Therefore, using the SUV_mean_ with PET/CT imaging could simplify the use of ^18^F-FDG in this human model for determining the efficacy of novel pulmonary anti-inflammatory treatments.

Other studies also demonstrate the importance of evaluating anti-inflammatory therapies in human models. Our results are in line with two other studies evaluating the effects of pioglitazone and zileuton on lung inflammation in healthy volunteers. In one study, 60 mg of pioglitazone administered daily for 9 days did not affect vascular responses in healthy volunteers after intravenous injection of endotoxin [44]. Another study using the same zileuton dosing regimen as employed in this study had no effect on exhaled nitric oxide measurements or peripheral blood neutrophilia in healthy volunteers after a 3 hour exposure to swine dust [45]. These results further contrast with the multiple studies in preclinical models demonstrating an anti-inflammatory effect of both drugs on neutrophilic inflammation [27–31, 33, 46]. Together, these studies highlight the value of using human models to study inflammatory responses to assess anti-inflammatory treatment efficacy.

Our results also contrast with clinical studies in patients demonstrating that long-term glitazone treatment reduces systemic inflammation. Treatment with either rosiglitazone (4–8 mg daily for 8 to 26 weeks) or pioglitazone (45 mg daily for 3 months) significantly reduced C-reactive protein (CRP) levels in patients with diabetes [47, 48] as well as in non-diabetic patients with coronary artery disease [49] and rheumatoid arthritis [50]. Obese, non-diabetic volunteers treated with 10 weeks of pioglitazone at 45 mg daily had reduced numbers of pro-inflammatory M1 macrophages in adipose tissue assessed by biopsy before and after treatment [51]. In contrast, our study demonstrated no effect on CRP levels as a result of two weeks of pioglitazone treatment, which could be due to the relatively short duration of pioglitazone treatment used in this study. This lack of effect could also be due to the fact that, in the model we used, the endotoxin induces acute inflammation that is resolved within 48 hours, in contrast to the chronic inflammation seen in these diseases. Nevertheless, studying acute endotoxin responses in humans may still be valuable as such studies will highlight how the human inflammatory response differs from that seen in animal models. Such data can guide the development of animal models that better reflect human inflammation biology and thus improve their utility for basic studies of lung inflammation as well as biomarker and drug development.

Several limitations must be considered for this study. One limitation was our inability to confirm that adequate drug levels were achieved in the airspaces. We were not able to detect group differences in the lipid mediators using mass spectrometry as we had expected. We cannot exclude the possibility that we did not achieve adequate levels of pioglitazone in the airways to have an effect. However, previous studies demonstrating that zileuton doses similar to our study reduced eosinophilic recruitment in asthma patients exposed to allergen [52, 53] suggest that our zileuton dosing was adequate. We did not control for the presence or absence of oropharyngeal sources of inflammation, such as dental disease, which could have promoted a more robust inflammatory response. We also did not control for differences in diet, which could have affected urinary LTE_4_ levels and limited our ability to detect differences among the groups [54, 55]. We excluded the possibility that any of the volunteers were on over-the-counter medications with anti-inflammatory properties at the time of entry into the study, limiting the possibility that these medications would affect the study results. Finally, we did not account for changes in blood volume on the ^18^F-FDG PET signal as we did not have an independent measure of blood volume. We have previously shown that, in this human model, treatment-induced reductions in ^18^F-FDG uptake can be detected [26] and that the BAL cells have higher ^18^F-FDG uptake than the BAL fluid by an order of magnitude [25]. These results suggest that, regardless of the impact of inflammation-induced blood volume changes, ^18^F-FDG uptake still reflects neutrophil recruitment, in part, and can still serve as a modifiable inflammatory marker in this model. Recently published quantitative models suggest that accounting for blood volume changes could change the interpretation of the ^18^F-FDG PET data (reviewed in [56]). However, since these models have not yet been validated with independent measures of blood volume, the impact of changes in blood volume on interpreting ^18^F-FDG uptake as marker of inflammation will require further study.

## Conclusion

In summary, we have shown that short courses of pioglitazone and zileuton have no effect on endotoxin-induced lung inflammation in healthy volunteers using ^18^F-FDG uptake, quantified as either *K*_i_ or the SUV_mean_. This result continues to support the utility of using ^18^F-FDG PET/CT imaging to measure the effects of anti-inflammatory drugs and to demonstrate the value of testing anti-inflammatory agents in humans before embarking on larger patient clinical trials.

## Supporting information

S1 Table Volumes of volumes of interest (VOI) drawn based on computed tomography (CT) images and the number of voxels contained each VOI when transferred to the positron emission tomography (PET) images. (DOCX)

S1 File Methods. Additional methods for capsule blinding, image analysis, and urine and bronchoalveolar lavage fluid assays.
(DOCX)

S2 File Adverse events results. Detailed listing of adverse events that occurred during the trial.
(DOCX)

S3 File Mass spectrometry results. (DOCX)

S4 File TREND statement checklist. (PDF)

S5 File Original approved protocol. The original intent was to test the anti-inflammatory effect of rosiglitazone.
(DOC)

S6 File Final approved protocol. Protocol under which this study was ultimately conducted. Since rosiglitazone became unavailable after the initial approval of this study, pioglitazone was used instead. This change is reflected in this protocol.
(DOC)

S7 File Institutional Review Board approval memo (approval-memo glitazone study 2012-02-08.rtf). (RTF)

S1 Dataset Data supplement. All source data that is summarized in the manuscript.
(XLSX)

## Abbreviations

^18^F-FDG: ^18^F-Fluorodeoxyglucose
ANOVA: Analysis of variance
ARDS: Acute respiratory distress syndrome
BAL: Bronchoalveolar lavage
COPD: Chronic obstructive pulmonary disease
CRP: C-reactive protein
CT: Computed tomography
ELISA: Enzyme-linked immunosorbent assay
ESR: Erythrocyte sedimentation rate
FEV1: Forced expiratory volume in 1 second
FVC: Forced vital capacity
i.b.: Intrabronchial
LTE4: Leukotriene E4
NIH: National Institutes of Health
PET: Positron emission tomography
PPAR: Peroxisome proliferator-activated receptor
SUV_mean_: Mean standard uptake value
TLR4: Toll-like receptor 4
