# Supplementary material for: The peroxisome proliferator-activated receptor agonist pioglitazone and 5-lipoxygenase inhibitor zileuton have no effect on lung inflammation in healthy volunteers by positron emission tomography in a single-blind placebo-controlled cohort study
Source: PLoS One. 2018 Feb 7;13(2):e0191783. doi: 10.1371/journal.pone.0191783 (PMC5802889; doi:10.1371/journal.pone.0191783)
Supplement: S1 Table — (DOCX) [file pone.0191783.s001.docx]

**S1 Table**

**Volumes of volumes of interest (VOI) drawn based on computed tomography (CT) images and the number of voxels contained each VOI when transferred to the positron emission tomography (PET) images.**

|  | **CT VOI volumes (cm^3^)** | | **PET voxel number within VOIs** | |
| --- | --- | --- | --- | --- |
| **Treatment Cohort** | **Left lung** | **Right lung** | **Left lung** | **Right lung** |
| **Placebo** | 20±18 | 35±20 | 357±327 | 553±411 |
| **Pioglitazone** | 20±7.3 | 20±7.7 | 378±221 | 627±348 |
| **Zileuton** | 21±12 | 31±23 | 348±129 | 531±136 |
