# Supplementary material for: The peroxisome proliferator-activated receptor agonist pioglitazone and 5-lipoxygenase inhibitor zileuton have no effect on lung inflammation in healthy volunteers by positron emission tomography in a single-blind placebo-controlled cohort study
Source: PLoS One. 2018 Feb 7;13(2):e0191783. doi: 10.1371/journal.pone.0191783 (PMC5802889; doi:10.1371/journal.pone.0191783)
Supplement: S1 File — Additional methods for capsule blinding, image analysis, and urine and bronchoalveolar lavage fluid assays. (DOCX) [file pone.0191783.s002.docx]

**METHODS**

**Encapsulation for blinding**

Zileuton tablets were overencapsulated with a size 000 gelatin capsule (Gallipot, Inc., St. Paul, MN) for blinding. Pioglitazone was overencapsulated with a size 00 capsule (Gallipot, Inc., St. Paul, MN) for blinding and cushioned with lactose monohydrate NF (Spectrum Co., Gardena, CA). Matching placebo pills containing lactose were also created.

**Image analysis**

To delineate the areas of interest for analysis, baseline PET/CT scans were co-registered to the post-endotoxin scan using Integrated Research Workflow 4.0 (Siemens). Volumes of interest (VOI) were placed on the post-endotoxin CT images over areas of infiltrate in the right lung and in the left lung at approximately the same anterior-posterior and cranial-caudal levels as the right lung VOI, adjusted to avoid the heart. For the *K*_i_, Patlak plots were generated for all volunteers using the time-activity curve determined from venous blood samples as well as the lung VOI time-activity curve from the PET images as previously described [[1](#_ENREF_1), [2](#_ENREF_2)]. The few small negative *K*_i_ values observed were set to zero. These were observed primarily in the left lung or in the baseline images (prior to endotoxin instillation).

**Urine creatinine determination**

Urine leukotriene E_4_ (LTE_4_) excretion was normalized to creatinine (pg/mg creatinine). Human urinary creatinine was determined by liquid chromatography-tandem mass spectrometry (LC-MS/MS) as follows: one µL of human urine was diluted 1mL of water containing 5 µg deuterated creatinine-d_3_ as the internal standard for LC-MS/MS analysis. Five point creatinine calibration standard samples containing the internal standard were also prepared for the absolute quantification. 1µL of both diluted urine and calibration samples was injected onto a C18 column (Atlantis 150x2.1 mm, 3µm) column connected to a Shimazdu 20AD HPLC system, interfaced with an API-4000 triple quadruple mass spectrometer for creatinine analysis. Acetonitrile and water containing 0.1% formic acid were used for chromatographic separation of creatinine. Positive ion electrospray multiple reaction monitoring (MRM) mode was controlled by Analyst software.

**Bronchoalveolar lavage (BAL) fluid assays**

Mass spectrometry was performed to assay for 5- and 15-hydroxyeicosatetraenoic acid (5-HETE and 15-HETE, respectively), leukotriene B_4_ (LTB_4_), and LTE_4_ in BAL fluid. Deuterated eicosanoids (lipid mediators) purchased from Cayman Chemical, 4 ng each of 5-HETE-d_8_, 15-HETE-d_8_, LTB_4_-d_4_, and LXA_4_-d_5_ as well as 10 ng of LTE_4_-d_5_, were spiked in each of BAL samples containing 70 % methanol. They were completely dried under a stream of nitrogen and were reconstituted with 500 µL of 1:1 methanol/water for LC-MS/MS analyses. At the same time, four point calibration samples for these eicosanoids were prepared in the presence of the deuterated internal standards for the absolute quantification.

Sample analysis was performed by the LC-MS/MS system described above. Fifty µL of each samples was injected onto a Thermo-Keystone HPLC column (2.x 100 mm, 3 µm), using solvent A (10 mM ammonium acetate in water) and solvent B (acetonitrile) at flow rate of 0.7 mL/min with a solvent gradient. The negative ion electrospray MRM mode was used for detection of both 5 endogenous eicosanoids and their deuterated internal standards. All study samples were injected in duplicate for data averaging. Data processing was conducted with Analyst 1.5.1.

The urea dilution assay was used to apply epithelial cell lining fluid corrections as previously described [[3](#_ENREF_3)].

**References**

1. Chen DL, Bedient TJ, Kozlowski J, Rosenbluth DB, Isakow W, Ferkol TW, et al. [18F]fluorodeoxyglucose positron emission tomography for lung antiinflammatory response evaluation. Am J Respir Crit Care Med. 2009;180(6):533-9. PubMed PMID: 19574441.

2. Chen DL, Rosenbluth DB, Mintun MA, Schuster DP. FDG-PET imaging of pulmonary inflammation in healthy volunteers after airway instillation of endotoxin. J Appl Physiol (1985). 2006;100(5):1602-9. Epub 2006/01/21. doi: 10.1152/japplphysiol.01429.2005. PubMed PMID: 16424067.

3. Rennard SI, Basset G, Lecossier D, O'Donnell KM, Pinkston P, Martin PG, et al. Estimation of volume of epithelial lining fluid recovered by lavage using urea as marker of dilution. J Appl Physiol. 1986;60(2):532-8. PubMed PMID: 3512509.
