# Supplementary material for: The peroxisome proliferator-activated receptor agonist pioglitazone and 5-lipoxygenase inhibitor zileuton have no effect on lung inflammation in healthy volunteers by positron emission tomography in a single-blind placebo-controlled cohort study
Source: PLoS One. 2018 Feb 7;13(2):e0191783. doi: 10.1371/journal.pone.0191783 (PMC5802889; doi:10.1371/journal.pone.0191783)
Supplement: S2 File — Detailed listing of adverse events that occurred during the trial. (DOCX) [file pone.0191783.s003.docx]

**S2 File**

**RESULTS**

**Adverse Events**

The majority of reported adverse events were expected and self-limited with none requiring specific intervention. The volunteer who had prolonged nausea after the first bronchoscopy did not require specific intervention for resolution of the nausea; however, the second bronchoscopy for bronchoalveolar lavage was canceled given this reaction. The following expected symptoms were reported during the study, all of which resolved without intervention (number of subjects reporting each symptom shown in parentheses): fever (N=2), cough (N=10), headache (N=7), sore throat (N=4), nausea (N=2, with one volunteer reporting prolonged, mildly severe nausea), constipation (N=1), upper respiratory infection (N=1), muscle/body aches (N=2), asymptomatic decreased hemoglobin and hematocrit (N=2), bruise and tenderness after intravenous catheter removal (N=1), shortness of breath (N=1), dizziness (N=1), and shoulder pain or soreness (N=1).
