# Supplementary material for: The peroxisome proliferator-activated receptor agonist pioglitazone and 5-lipoxygenase inhibitor zileuton have no effect on lung inflammation in healthy volunteers by positron emission tomography in a single-blind placebo-controlled cohort study
Source: PLoS One. 2018 Feb 7;13(2):e0191783. doi: 10.1371/journal.pone.0191783 (PMC5802889; doi:10.1371/journal.pone.0191783)
Supplement: S3 File — (DOCX) [file pone.0191783.s004.docx]

**S3 File**

**RESULTS**

**Mass spectrometry demonstrates no differences in lipid mediators in BAL fluid**

Two BAL fluid samples in the pioglitazone cohort and one in the placebo cohort could not be analyzed by mass spectrometry as a result of storage issues. The volunteer in the placebo cohort who did not undergo the BAL procedure had no BAL sample to analyze. Therefore, the following numbers of samples were processed for LTB_4_ and LTE_4_: N=4 pioglitazone, N=6 zileuton, and N=4 placebo cohort. These results showed no differences in levels among the cohorts after normalizing for the epithelial cell lining fluid based on a one-way analysis of variance (see Table below for summary data, p = 0.55 for LTB_4_, p = 0.15 for LTE_4_). Given the negative results using the primary outcome measure, *K*_i_, limited mass spectrometry testing of four samples taken from the pioglitazone cohort and two samples from the zileuton cohort was performed for 5-HETE, 15-HETE, and LXA_4_. These measurements showed low levels; therefore, no additional correction for epithelial cell lining fluid was applied, and no statistical testing was performed. These data are included in the data file supplement for this manuscript.

| **Treatment Cohort** | **LTB_4_** | **LTE_4_** |
| --- | --- | --- |
| Placebo | 0.099±0.046 | 0.73±0.73 |
| Pioglitazone | 0.11±0.10 | 0.32±0.34 |
| Zileuton | 0.064±0.027 | 0.16±0.13 |
