# Supplementary material for: The peroxisome proliferator-activated receptor agonist pioglitazone and 5-lipoxygenase inhibitor zileuton have no effect on lung inflammation in healthy volunteers by positron emission tomography in a single-blind placebo-controlled cohort study
Source: PLoS One. 2018 Feb 7;13(2):e0191783. doi: 10.1371/journal.pone.0191783 (PMC5802889; doi:10.1371/journal.pone.0191783)
Supplement: S5 File — The original intent was to test the anti-inflammatory effect of rosiglitazone. (DOC) [file pone.0191783.s006.doc]

Noninvasive quantification of the pulmonary anti-inflammatory effect of the glitazones

**Date:** December 12, 2011

**Version:** 1.4

Principal Investigator

Delphine L. Chen, MD

Co-Investigators

Warren Isakow, MD

Derek E. Byers, MD, PhD

Howard J. Huang, MD

Collaborators

Mario Castro, MD

Thomas W. Ferkol, MD

J. Philip Miller, PhD

Richard A. Pierce, PhD

Daniel B. Rosenbluth, MD

# SUMMARY

**Rationale**

Inflammation contributes to the development of a number of acute and chronic lung diseases, include acute lung injury/acute respiratory distress syndrome, chronic obstructive pulmonary disease, asthma, and cystic fibrosis. Therefore, novel drugs that can effectively reduce lung inflammation can potentially protect the lung from being destroyed and thus improve the outcomes of patients with these diseases. There is growing evidence that the peroxisome proliferator-activated receptor-γ (PPAR-γ) agonists, or glitazones, may be effective in reducing lung inflammation. However, the efficacy of this class in reducing lung inflammation has not been demonstrated in humans. There is also evidence that interaction with another known anti-inflammatory drug class, 5-lipoxygenase (5-LO) inhibitors (of which zileuton is clinically available) may interfere with the anti-inflammatory effect of the glitazones. We have successfully used a noninvasive imaging approach, positron emission tomography with [^18^F]fluorodeoxyglucose (FDG-PET), to quantify changes in experimentally-induced lung inflammation in healthy volunteers in response to anti-inflammatory therapy. Therefore, we propose to conduct a single-blind, placebo-controlled trial to determine whether pioglitazone is effective as an anti-inflammatory agent and whether zileuton will interfere with this effect. We will use FDG-PET to measure how well the drug treatments (pioglitazone alone, zileuton alone, and pioglitazone plus zileuton) reduce endotoxin-mediated lung inflammation in healthy volunteers. This trial will yield important and clinically relevant information regarding a new therapeutic drug class for lung inflammation. It will demonstrate the potential for using FDG-PET as a clinically useful biomarker for quickly assessing whether a novel drug is effective in reducing lung inflammation.

**Objective**

The primary objective of this study is to demonstrate that pioglitazone effectively suppresses endotoxin-mediated lung inflammation in healthy volunteers, using FDG-PET imaging as the biomarker for lung inflammation.

**Trial Design**

This is a single center, prospective, single-blind placebo-controlled clinical trial. Twenty-four healthy volunteers will be recruited sequentially into the following four treatment groups in this order: pioglitazone plus oral placebo, zileuton plus oral placebo, pioglitazone plus zileuton, or two oral placebos. All volunteers will be blinded to the study drugs. They will then receive endotoxin instilled bronchoscopically in the right middle lobe. Enrolled volunteers will receive their assigned drug treatment as an outpatient and then undergo a baseline FDG-PET scan on Admission Day 1, receive endotoxin on Admission Day 2, and undergo a post-endotoxin FDG-PET scan followed by bronchoalveolar lavage (BAL) on Admission Day 3.

**Endpoints**

Primary Endpoints:

- Change in *K*_i_, the influx constant that describes the rate of [^18^F]FDG uptake into the right middle lobe, between baseline and post-endotoxin scans
- Absolute *K*_i_ after endotoxin

Secondary Endpoints:

- BAL cell counts (total and neutrophil counts)
- BAL PPAR- γ transcriptional activity
- BAL LXA_4_, LTB_4_, IL-8 and TNF-α levels (measured by ELISA)
- BAL 5-LO, 15-LO, PPAR-γ mRNAlevels
- Urine LTE_4_ levels.

Exploratory Endpoints:

- CT density (in Hounsfeld Units, HU)
- Airway thickness
- Respiratory-gated standard uptake value (SUV)
- Presence of Toll-like receptor 4 (TLR4) polymorphisms

**Statistical Methods**

An interim analysis will be performed after the first treatment group is filled. If there is a minimum of a 30% drop in the post-endotoxin *K*_i_ from the baseline *K*_i_, enrollment will continue into the next 3 groups. If no change in *K*_i_ is observed at this interim analysis, we will continue to enroll volunteers into the zileuton+placebo and placebo+placebo groups but will not enroll volunteers into the pioglitazone+zileuton group. We recognize that this approach reduces the power of the study but felt this was justified as we would still be able to adequately test our hypotheses while minimizing the risks to the volunteers for this study. At the end of the study, a three-way mixed model analysis of variance (zileluton, pioglitazone, and time ANOVA) will be used to assess the effect of the drug treatments on change in *K*_i_ from baseline and *K*_i_ after treatment, the primary endpoints among the treatment groups. Appropriate prespecified contrasts will be used for the primary tests. An exploratory analysis using a two-way ANOVA will also be performed to analyze the treatment effects on the BAL total cell counts, BAL neutrophil counts, PPAR-γ transcriptional activity, LXA_4_, LTB_4_, IL-8, and TNF-α levels (by ELISA) in the BAL fluid, and 5-LO, 15-LO and PPAR-gamma mRNA levels in BAL cells, although this study will not be powered specifically to detect these relationships. Correlations between the post-endotoxin *K*_i_ and the BAL and urine variables will be performed using standard linear regression with calculation of the coefficient of determination (R^2^). Correlations will also be explored among serum and BAL measurements and *K*_i_. Any volunteer with incomplete scan data will not be included in the analysis. Any other missing data (i.e. missing BAL data point) will be handled by excluding that data point from that particular analysis. We will also assure that no bias was introduced by this handling of the missing data by using multiple imputation for all missing data.

# Table of Protocol-Required Procedures

| **PROCEDURE** | **Screen** | **Outpatient Drug Treatment (2 weeks)** | **Day 1 (Inpatient)** | **Day 2**  **(Inpatient)** | **Day 3**  **(Inpatient)** | **Day 4** | **Day 10 + 2 d** |
| --- | --- | --- | --- | --- | --- | --- | --- |
| Inclusion/Exclusion criteria | X |  |  |  |  |  |  |
| Informed consent | X |  |  |  |  |  |  |
| Height/weight/VS | X |  |  |  |  |  |  |
| History and physical exam | X |  |  |  |  |  |  |
| Chest X-ray | X |  |  |  |  |  |  |
| Electrocardiogram (ECG) | X |  |  |  |  |  |  |
| Pulmonary function test (PFT) | X |  |  |  | X |  |  |
| Blood work: CBC, CMP, CRP, ESR, PT/PTT* | X |  | X |  | X |  |  |
| Blood sample for research lab assays | X |  | X |  | X |  |  |
| Urine sample for research lab assays | X |  | X |  | X |  |  |
| Urinalysis | X |  |  |  |  |  |  |
| Serum hCG | X |  |  |  |  |  |  |
| Urine hCG |  |  | X |  |  |  |  |
| Pioglitazone+placebo, zileuton+placebo, pioglitazone+zileuton, or placebo+placebo |  | X | X | X | X |  |  |
| Medication diary |  | X |  |  |  |  |  |
| FDG-PET scan |  |  | X |  | X |  |  |
| Sulfa vs amoxicillin |  |  | X | X |  |  |  |
| Nothing by mouth after breakfast |  |  | X |  |  |  |  |
| Nothing by mouth after midnight |  |  | X | X |  |  |  |
| Bronchoscopy |  |  |  | X | X |  |  |
| Endotoxin instillation |  |  |  | X |  |  |  |
| Bronchoalveolar lavage (BAL) |  |  |  |  | X |  |  |
| Phone call |  | X |  |  |  | X | X |

* Not repeated on Day 3.

# ABBREVIATION LIST

| [^18^F]FDG: [^18^F]fluorodeoxyglucose |
| --- |
| [^3^H]DG: [^3^H]deoxyglucose |
| 5-LO: 5-lipoxygenase |
| 15-LO: 15-lipoxygenase |
| 15d-PGJ_2_: 15-deoxy-∆^12,14^ prostaglandin J_2_ |
| 15S-HETE: 15*S*-hydroxyeicosatetraenoic acid |
| ALI: Acute lung injury |
| ANOVA: Analysis of variance |
| ARDS: Acute respiratory distress syndrome |
| BAL: Bronchoalveolar lavage |
| BMI: Body mass index |
| BOS: Bronchiolitis obliterans syndrome |
| CBC: Complete blood count |
| CCIR: Center for Clinical Imaging Research |
| CF: Cystic fibrosis |
| CFR: Code of Federal Regulations |
| CMP: Complete metabolic profile |
| COPD: Chronic obstructive pulmonary disease |
| CRP: C-reactive protein |
| CRU: Clinical Research Unit |
| CT: Computed tomography |
| DSMB: Data Safety Monitoring Board |
| ECG: electrocardiogram |
| ESR: Erythrocyte sedimentation rate |
| FDA: Food and Drug Administration |
| FDG-PET: Positron emission tomographic imaging with [^18^F]fluorodeoxyglucose |
| FEV_1_: Forced expiratory volume in 1 second |
| FVC: Forced vital capacity |
| GCRC: General Clinical Research Center |
| hCG: Urine human chorionic gonadotropin |
| i.v.: intravenous |
| ID: Identification |
| IL-8: Interleukin-8 |
| IND: Investigational New Drug |
| IRB: Institutional Review Board |
| *K*_i_: Influx constant determined by Patlak graphical analysis |
| LTB_4_: Leukotriene B_4_ |
| LTE_4_: Leukotriene E_4_ |
| LXA_4_: Lipoxin A_4_ |
| mRNA: Messenger ribonucleic acid |
| NIH: National Institutes of Health |
| NPO: Nil per os (nothing by mouth) |
| PET: Positron emission tomography |
| PFT: Pulmonary function test |
| PPAR-γ: Peroxisome proliferator-activated receptor-γ |
| PT: Prothrombin time |
| PTT: Partial thromboplastin time |
| RDRC: Radioactive Drug Research Committee |
| SAE: Serious adverse event |
| SUV: Standard uptake value |
| TLR4: Toll-like receptor 4 |
| TNF-α: Tumor necrosis factor α |
| VS: Vital signs |
|  |

TABLE OF CONTENTS

[SUMMARY 2](#__RefHeading___Toc281916596)

[Table of Protocol-Required Procedures 4](#__RefHeading___Toc281916597)

[ABBREVIATION LIST 5](#__RefHeading___Toc281916598)

[1. INTRODUCTION 8](#__RefHeading___Toc281916599)

[1.1. Background and Significance 8](#__RefHeading___Toc281916600)

[1.2. Specific Aim 11](#__RefHeading___Toc281916601)

[2. STUDY METHODS 11](#__RefHeading___Toc281916602)

[2.1. Study Design 11](#__RefHeading___Toc281916603)

[2.2. Study Endpoints 12](#__RefHeading___Toc281916604)

[3. SUBJECTS 13](#__RefHeading___Toc281916605)

[3.1. Inclusion Criteria 13](#__RefHeading___Toc281916606)

[3.2. Exclusion Criteria 13](#__RefHeading___Toc281916607)

[3.3. Women of Child-Bearing Potential 13](#__RefHeading___Toc281916608)

[3.4. Number of Subjects 14](#__RefHeading___Toc281916609)

[3.5. Subject Withdrawal and Replacement 14](#__RefHeading___Toc281916610)

[4. STUDY PROCEDURES 14](#__RefHeading___Toc281916611)

[4.1. Screening 14](#__RefHeading___Toc281916612)

[4.2. Outpatient Drug Treatment 14](#__RefHeading___Toc281916613)

[4.3. Bronchoscopic Administration of Endotoxin and Bronchoalveolar Lavage 16](#__RefHeading___Toc281916614)

[4.4. PET-CT Imaging with [^18^F]FDG 17](#__RefHeading___Toc281916615)

[4.5. Blood Collection 17](#__RefHeading___Toc281916616)

[4.6. Videotaping of Bronchoscopy Procedures 17](#__RefHeading___Toc281916617)

[4.7. Specific Procedures Timeline 18](#__RefHeading___Toc281916618)

[4.8. Criteria for discharge from the CRU 20](#__RefHeading___Toc281916619)

[5. ADVERSE EVENT REPORTING AND DATA/SAFETY MONITORING 21](#__RefHeading___Toc281916620)

[5.1. Definitions 21](#__RefHeading___Toc281916621)

[5.2. Causality of adverse events 21](#__RefHeading___Toc281916622)

[5.3. Safety Monitoring 21](#__RefHeading___Toc281916623)

[5.4. Collecting/reporting of adverse events 22](#__RefHeading___Toc281916624)

[5.5. Follow-up of serious adverse events 23](#__RefHeading___Toc281916625)

[5.6. Potential Risks 23](#__RefHeading___Toc281916626)

[5.7. Adequacy of Protection Against Risks 27](#__RefHeading___Toc281916627)

[6. DATA ANALYSIS/STATISTICAL METHODS 29](#__RefHeading___Toc281916628)

[6.1. Sample Size Calculation 29](#__RefHeading___Toc281916629)

[6.2. Statistical Analysis 29](#__RefHeading___Toc281916630)

[7. STUDY ADMINISTRATION 30](#__RefHeading___Toc281916631)

[7.1. direct Access to Source Data and Documents 30](#__RefHeading___Toc281916632)

[7.2. Ethics 30](#__RefHeading___Toc281916633)

[7.3. Records Retention and Data Archival 31](#__RefHeading___Toc281916634)

[REFERENCES 31](#__RefHeading___Toc281916635)

[APPENDIX 36](#__RefHeading___Toc281916636)

# INTRODUCTION

Inflammation contributes to the development of a number of acute and chronic lung diseases. Effective treatments that decrease lung inflammation are needed to improve patient outcomes. PPAR-γ agonists such as rosiglitazone and pioglitazone effectively reduce lung inflammation in animal models. However, the specific lung anti-inflammatory effect of this drug class *in humans* has not been evaluated. We have developed a method for assessing the effect of anti-inflammatory therapies specifically on lung inflammation that can be used to test the efficacy of the glitazones as an anti-inflammatory drug. We systematically evaluated the ability of FDG-PET to quantify lung inflammation, beginning with a pre-clinical canine model of acute lung injury and then applying this imaging method to a human model of experimentally-induced lung inflammation.

Our goal is to now use our approach to determine whether the PPAR-γ agonists are a potentially effective drug class for treating inflammatory lung disease. We will test the overall hypothesis that pioglitazone, a PPAR-γ agonist, effectively suppresses endotoxin-mediated lung inflammation and that 5-lipoxygenase (5-LO) inhibition reverses this effect by accomplishing the following single Specific Aim. The results from this clinical trial will yield important information regarding whether the PPAR-γ agonists are potentially effective agents for treating inflammatory lung disease. We will also demonstrate the potential clinical value of using FDG-PET imaging to quantify the response of lung inflammation to treatment.

## Background and Significance

### Neutrophilic inflammation as a therapeutic target in acute and chronic inflammatory lung diseases

A number of acute and chronic lung diseases, including acute respiratory distress syndrome (ARDS), cystic fibrosis (CF)[^3-5^](#_ENREF_3), chronic obstructive pulmonary disease (COPD)[^6-9^](#_ENREF_6), sub-types of asthma, and post-lung transplant bronchiolitis obliterans syndrome (BOS), are characterized by persistent neutrophilic inflammation. Given that dysregulated neutrophil activation is thought to cause continued oxidant and protease production, ultimately destroying normal lung tissues, identifying novel therapeutics to reduce lung inflammation is an important area of drug development.

- - 1. **New tools for quantifying pulmonary inflammation: the promise of *in vivo* imaging**

PET imaging with [^18^F]FDG is a noninvasive approach for quantifying the degree of lung inflammation that overcomes some of the limitations of current biomarkers for lung inflammation. Bronchoalveolar lavage (BAL), considered the gold standard for documenting lung inflammation, is an invasive procedure and subject to sampling errors as only a few segments of the lung can be sampled at one time. Induced sputum, while noninvasive, is dependent on patient effort to obtain an adequate sample. PET, on the other hand, is quantitative and highly sensitive, requiring only nanomolar or fentomolar concentrations of tracer to generate a detectable signal without perturbing the metabolic processes being measured[^18^](#_ENREF_18). In addition, PET can image the entire lung (that which is in the field-of-view), and its accuracy is not dependent on patient effort. Multiple clinical studies have demonstrated that FDG-PET can quantify varying levels of inflammation in patients with asthma[^19^](#_ENREF_19), pneumonia[^20^](#_ENREF_20), COPD[^21^](#_ENREF_21), and cystic fibrosis. FDG-PET also appears to be capable of detecting clinically relevant changes in lung inflammation within the same patient more effectively than measures such as pulmonary function tests. We and others have demonstrated that the [^18^F]FDG is taken up by activated neutrophils and that it correlates strongly with the enzyme activity of the neutrophil-specific tissue concentration of myeloperoxidase[^25^](#_ENREF_25) and with the number of neutrophils present in BAL fluid. We have also shown that in cells isolated from BAL fluid, the *in vitro* uptake of another glucose analog, ^3^H-deoxyglucose ([^3^H]DG), is limited to neutrophils. Finally, the pulmonary parenchyma may also contribute to [^18^F]FDG uptake[^25^](#_ENREF_25). *These data indicate that FDG-PET is a clinically usable imaging technique by which we can quantify the total lung inflammatory burden in vivo.*

Despite this potential clinical application of FDG-PET, there are several limitations to consider. First, [^18^F]FDG is an analog of glucose; therefore, FDG-PET essentially measures glucose uptake. Changes in glucose uptake may not be tied mechanistically to a drug’s particular mode of action. Despite this, the fact that FDG-PET measures a downstream effect of an anti-inflammatory treatment may instead be a strength, as it is less likely to miss a real anti-inflammatory effect[^24^](#_ENREF_24). Additionally, the radiation dose to patients must be taken into consideration, particularly if this technique is to be used in children. Careful thought must be given to any clinical implementation of FDG-PET imaging to ensure that information obtained by PET clearly outweighs any risk from the cumulative radiation dose to the patients.

- - 1. **Models for studying lung inflammation in humans**

Clinical trials are essential for demonstrating the efficacy of therapeutics *in patients.* However, these trials can be expensive and time-consuming to complete. Testing the efficacy of drugs under development in rodent models also does not always translate to success in clinical trials. Therefore, identifying *a human model* which can be used to more reliably predict the efficacy of a putative treatment on airway inflammation in patients would be ideal. In the sepsis field, testing the effects of potentially useful immunomodulatory drugs in normal volunteers after the intravenous infusion of small doses of endotoxin before embarking upon a larger study in actual patients is the current standard practice[^28-34^](#_ENREF_28). Although previously not done, an analogous platform for testing therapies targeting pulmonary inflammation would be highly advantageous[^18^](#_ENREF_18).

Several years ago,[^35^](#_ENREF_35) investigators at the NIH showed that focal, limited, airway and surrounding parenchymal inflammation could be safely induced by the direct bronchial instillation (intrabronchial, i.b.) of small amounts of endotoxin into the airway of a single lung segment in 20 healthy non-smoking human research volunteers. Neutrophil concentrations in the BAL from challenged lung segments increased by 32-fold 24 h after endotoxin instillation but were back to near control values by 48 h. Since the publication, the model has been employed in an additional 154 subjects, described in 7 additional publications, including two by our own group. To date, there have been no unexpected adverse events associated with use of this model. The potential clinical relevance of this model is supported by a study showing that proteomic patterns in BAL recovered after endotoxin instillation were similar to those identified in 11 patients with ARDS.[^41^](#_ENREF_41)

Accordingly, we are the first group to develop a noninvasive imaging approach that can quantify the anti-inflammatory effect of a drug in a human model of experimentally-induced lung inflammation[^37^](#_ENREF_37). We validated the application of FDG-PET imaging in a canine model of acute lung injury. We next used this imaging approach to quantify experimentally-induced lung inflammation in healthy volunteers in a model first characterized at the NIH[^35^](#_ENREF_35). Finally, we successfully used this human model of lung inflammation to demonstrate that lovastatin effectively suppresses the lung’s inflammatory response using FDG-PET as the primary outcome measure[^37^](#_ENREF_37). Therefore, *we are uniquely qualified to study the effect of novel anti-inflammatory treatments on the levels of neutrophil activation and recruitment as measured by FDG-PET*.

### 1.1.4. The potential role of PPAR-γ agonists in ameliorating the inflammatory process

There still remains a great need for identifying effective therapies for patients with inflammatory lung disease. Emerging evidence suggests that activation of PPAR-γ may represent a novel approach for treating lung inflammation[^43-46^](#_ENREF_43). High doses of the PPAR-γ ligand 15-deoxy-∆^12,14^ prostaglandin J_2_ (15d-PGJ_2_) cause macrophages to convert to a resting phenotype as a result of PPAR-γ-antagonism of the pro-inflammatory transcription factors AP-1, STAT, and nuclear factor-κB (NFκB)[^47^](#_ENREF_47). Upregulation of PPAR-γ in NIH-A549 airway epithelial cells leads to decreased production of interleukin-8 (IL-8), a potent neutrophil chemoattractant and activator[^48^](#_ENREF_48). PPAR-γ activation in neutrophils decreases their chemotactic response to IL-8[^49^](#_ENREF_49). These data suggest that increasing the expression of PPAR-γ in the airways may help ameliorate the intensity of the lungs’ inflammatory response by reducing both activation of and cytokine production by macrophages and airway epithelial cells and reducing neutrophil migration.

Thiazolidinediones, or glitazones, are PPAR-γ agonists that have been used successfully for the treatment of diabetes. Given the potential anti-inflammatory properties of PPAR-γ as described above, these drugs have been suggested as a novel approach for treating inflammatory lung disease. Rosiglitazone treatment in models of endotoxin-induced lung injury in mice[^50^](#_ENREF_50) and rats[^51^](#_ENREF_51), and carageenan-induced pleurisy in rats effectively reduced neutrophil recruitment, edema formation, and lung injury. However, no in vivo data are available regarding the mechanisms mediating this anti-inflammatory effect.

Studies performed in other organ systems suggest a possible mechanism which we can test in our human model of lung inflammation. Lipoxin A_4_ (LXA_4_) is an endogenous eicosanoid that promotes the resolution of inflammation[^54^](#_ENREF_54). LXA_4_ can be produced by the action of 5-LO in neutrophils on the 15-lipoxygenase (15-LO) metabolite of arachidonic acid, 15*S*-hydroxyeicosatetraenoic acid (15S-HETE)[^54^](#_ENREF_54). Administration of stable LXA_4_ analogs causes downregulation of IL-8 production in intestinal epithelial cells exposed to *Salmonella typhimurium*[^55^](#_ENREF_55), similar to the effect of PPAR-γ expression in airway epithelial cells[^48^](#_ENREF_48). A recent study demonstrated in a rat model of stroke that rosiglitazone increased expression of 5-LO mRNA in neurons and LXA_4_ production in the brain cortex while inhibiting production of leukotriene B_4_ (LTB_4_), a potent chemoattractant for neutrophils[^56^](#_ENREF_56). Interestingly, while treatment with a potent 5-LO inhibitor BWA4C[^57^](#_ENREF_57) as a single agent also reduced LTB_4_ production in this model, giving BWA4C with rosiglitazone actually inhibited the LXA_4_ production induced by rosiglitazone[^56^](#_ENREF_56). BWA4C treatment also reversed rosiglitazone-mediated neuroprotection and suppression of cyclooxygenase-2 (COX-2), iNOS, and the mature form of tumor necrosis factor-α (TNF- α)[^56^](#_ENREF_56). Given these data, we hypothesize that increased PPAR-γ may promote suppression or resolution of lung inflammation through increased production of LXA_4_ by upregulating 5-LO activity in alveolar macrophages and airway epithelial cells (which have been shown to express 5-LO[^58^](#_ENREF_58)).

Pioglitazone, another PPAR-γ agonist, has also been effective in decreasing lung inflammation in animal models. Pioglitazone also promotes the production of the anti-inflammatory 15-epi-lipoxin A_4_ (15-epi-LXA_4_) by 5-lipoxygenase in cardiac myocytes, suggesting that its anti-inflammatory effect is also tied to 5-lipoxygenase expression.

Based on the above, we plan to test the hypotheses *in humans* that 1) pioglitazone is effective in reducing endotoxin-mediated inflammation, and 2) that zileuton, a 5-LO inhibitor, will paradoxically block the anti-inflammatory effect of pioglitazone. We recognize that zileuton is an effective anti-inflammatory drug in patients with asthma[^63^](#_ENREF_63) and has effectively reduced endotoxin-mediated inflammation in rodent models. Pioglitazone and zileuton together may therefore synergistically suppress inflammation. Thus, the hypothesis that zileuton might interfere with the anti-inflammatory effect of another drug is counterintuitive. However, the data in the rat stroke model demonstrating decreased LTB_4_ production with 5-LO inhibition alone but abolition of anti-inflammatory effects with combined 5-LO inhibition and PPAR-γ activation are compelling[^56^](#_ENREF_56). If our results support our hypothesis, these findings will underscore the importance of understanding clearly the mechanisms by which these drugs operate in humans to ensure that combinatorial drug therapy can truly be optimized for the patient.

## Specific Aim

Specific Aim: Perform a prospective, single-blind, placebo-controlled trial to evaluate the effectiveness of the PPAR-γ agonist pioglitazone in reducing endotoxin-mediated neutrophilic inflammation in healthy volunteers, using PET-measured [^18^F]FDG uptake, measured by *K*_i_ (the influx constant calculated by Patlak graphical analysis), as the primary outcome measure of efficacy.

The following hypotheses will be tested in this clinical trial:

1. Pioglitazone as a single agent effectively suppresses endotoxin-mediated lung inflammation in healthy volunteers, leading to a significant decrease in *K*_i_ relative to placebo treatment.
2. Zileuton, a 5-LO inhibitor, as a single agent effectively suppresses endotoxin-mediated lung inflammation in healthy volunteers, leading to a significant decrease in *K*_i_ relative to placebo treatment.
3. Effective inhibition of 5-LO by zileuton reverses the anti-inflammatory effect of pioglitazone on endotoxin-mediated lung inflammation. Combined pioglitazone and zileuton therapy will lead to a significant increase in *K*_i_ relative to pioglitazone treatment alone.
4. **STUDY METHODS**
   1. **Study Design**

This is a single-center, prospective, single-blind, placebo-controlled trial to evaluate the effectiveness of the PPAR-γ agonist pioglitazone in reducing endotoxin-mediated neutrophilic inflammation in healthy volunteers. All volunteers recruited to this study will be enrolled sequentially into the drug treatment groups being studied (receiving the drug treatment as an outpatient) and then undergo the same procedures upon admission. The procedure flow is illustrated in Figure 1.

**Figure 1.** Study Procedures


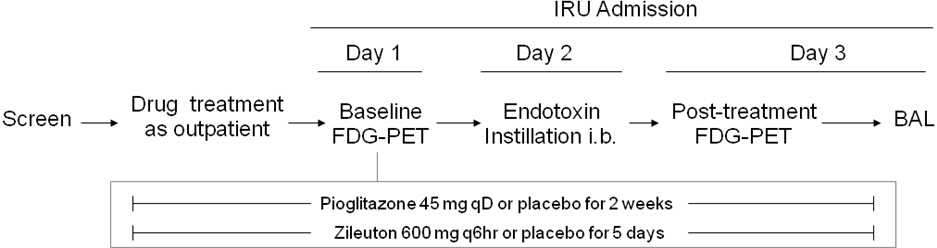


We have chosen this study design because: 1) There are no data in humans demonstrating that the dose of pioglitazone we intend to use in this trial is effective in reducing the endotoxin-mediated lung inflammation observed in this study; 2) While we believe the risks of administering both pioglitazone and zileuton are minimal given the rapid metabolism of each by different P450 enzymes in the liver, we believe this study design will allow us to better monitor for serious adverse events as they occur and, in the event that the dose of pioglitazone is not effective, will enable us to eliminate the pioglitazone+ziletuon treatment group and thus avoid unnecessarily exposing additional volunteers.

Volunteers will be enrolled sequentially into the following groups (in order): pioglitazone plus oral placebo, zileuton plus oral placebo, pioglitazone plus zileuton, or two oral placebos. All volunteers will be blinded to the study drugs they are receiving.

- 1. **Study Endpoints**

**Primary Endpoints**

- Change in *K*_i_, the influx constant that describes the rate of [^18^F]FDG uptake into the right middle lobe, between baseline and post-endotoxin scans.
- Absolute *K*_i_ after endotoxin

**Secondary Endpoints**

- BAL cell counts (total and neutrophil counts)
- BAL PPAR- γ transcriptional activity
- BAL LXA_4_, LTB_4_, IL-8 and TNF-α levels (measured by ELISA)
- BAL 5-LO, 15-LO, PPAR-γ mRNA levels
- Urine LTE_4_ levels

**Exploratory Endpoints**

- CT density (in Hounsfeld Units, HU)
- Airway thickness
- Respiratory-gated standard uptake value (SUV)
- Presence of TLR4 polymorphisms

1. **SUBJECTS**
   1. **Inclusion Criteria**

- Healthy man or woman, any race or ethnicity, age 19 – 44 years old
- Screening FEV_1_ and FVC > 90% of predicted
- Screening oxygen saturation by pulse oximetry >97% on room air
- Capable of lying still and supine within the PET/CT scanner for ~1.5 hours
- Capable of following instructions for breathing protocol during CT portion of PET/CT
- Able and willing to give informed consent
- BMI < 35
  1. **Exclusion Criteria**
- Pregnancy (confirmed by qualitative urine hCG pregnancy test)
- Lactation
- Active menstruation
- History of cardiopulmonary disease
- Currently taking any prescription medications
- History of tobacco use or illicit drug use within the past year
- Presence of implanted electronic medical device
- Enrollment in another research study of an investigational drug
- Known allergy to pioglitazone or zileuton
- Known allergy to both trimethoprim/sulfamethoxazole and amoxicillin
- Known allergy to drugs routinely used during bronchoscopy
- History of chronic active liver disease or acute liver disease within the past 3 months
- SGOT >47 IU/L, SGPT > 53 IU/L, or bilirubin > 1.1 mg/dl
- Inability to lie flat for 1.5 hours for PET/CT scans or follow breathing protocol instructions for the CT portion of the PET/CT
- Prior research-related radiation exposure within the past year such that participation in this study would result in exposures that exceed the limits as defined by the FDA RDRC regulations (21 CFR 361.1)
  1. **Women of Child-Bearing Potential**

We will confirm pregnancy test results in all women of child-bearing potential screened for this study. Women who meet the following criteria may also be enrolled without a screening pregnancy test:

- Post-menopausal status defined as women who are 40 years of age or older and have been amenorrheic for at least 2 years
- Women who have had a hysterectomy and/or bilateral oophorectomy
- Women who have been surgically sterilized with no unintended pregnancies for at least 2 years after the procedure
  1. **Number of Subjects**

We plan to screen 39 healthy volunteers to enroll 29 volunteers total. Based on our prior experience with this protocol, we anticipate that ~37% of those screened will not be enrolled either due to unwillingness to participate or failure of screening procedures. After enrollment, we anticipate an 18% dropout rate. Therefore, we plan to screen 39 people total, anticipating that 29 will pass the initial screening tests and proceed with study procedures, and 5 will drop out after beginning study procedures, leaving 24 who complete the protocol. This will give us N=6 per group. Enrollment will be stopped when the requisite number of volunteers is recruited.

- 1. **Subject Withdrawal and Replacement**

Subjects who do not complete the protocol-required procedures in their entirety will be replaced to ensure adequate evaluable data are obtained. Subjects are free to withdraw from the study at any time for any reason. Attempts will be made to discuss with the subject the reasons for withdrawal and to continue with the planned study evaluations, with the subject’s consent. No subject will be enrolled twice in this trial.

1. **STUDY PROCEDURES**
   1. **Screening**

All volunteers who give informed consent will undergo a physical exam and have heart rate, blood pressure, respiratory rate, and body temperature recorded. Bloodwork, including complete blood counts, routine chemistries, liver function tests, and coagulation parameters, a urinalysis, and a serum pregnancy test will be obtained. Blood and urine samples will also be obtained for research lab assays. Chest radiography, electrocardiogram and pulmonary function tests (PFTs) will also be obtained. Anyone with any history of cardiopulmonary disease will be excluded.

## Outpatient Drug Treatment

Each volunteer will take the following:

- Pioglitazone 45 mg or matching placebo by mouth once daily for 2 weeks prior to admission.
- Zileuton 600 mg or matching placebo by mouth with food qid for 5 days prior to admission.
  - 1. Justification for Dosing

There are no data indicating what dosing regimen for pioglitazone would be optimal for specifically reducing lung inflammation in humans. Several clinical trials in patients with diabetes have demonstrated decreases in human serum C-reactive protein levels with pioglitazone treatment (45 mg/day) for 8 weeks or longer. One study evaluated the ability of pioglitazone to reduce inflammatory markers and vascular changes in response to systemic endotoxin administration in healthy volunteers[^68^](#_ENREF_68). This particular study did not report any significant impact on TNF-alpha or IL-6 production in the blood at 2 or 4 hours after endotoxin in healthy volunteers who had been taking 60 mg of pioglitazone daily for 9 days. Since pioglitazone serum levels reach steady-state only after 7 days of drug administration, it is possible that the volunteers did not take the drug for a long enough period of time to have an anti-inflammatory effect. We also know that responses in the lungs to endotoxin may differ from that in the systemic circulation[^69^](#_ENREF_69), so while this study provides a benchmark for determining the dose for this study, the results may not be reflective of what would happen in the lungs after endotoxin exposure. Two studies have reported 9-day dosing in healthy volunteers at 45 and 60 mg per day without any serious side effects. Clinical trials of 8 and 12 weeks’ duration have been conducted in patients with diabetes with pioglitazone without serious side effects attributable to the drug. Based on the available information we have about pioglitazone, we have chosen to give volunteers 45 mg daily for 2 weeks for this particular study.

For zileuton, pharmacokinetic studies have shown that q6hr dosing of the immediate-release tablets achieves the best levels of drug over time[^71^](#_ENREF_71). One study demonstrated that LTB_4_ production was completely inhibited after swine dust exposure in healthy volunteers treated with 600 mg q6hr of zileuton for 5 days prior to exposure[^72^](#_ENREF_72). Therefore, we will use this same dosing regimen for 5 days prior to endotoxin instillation. We do not expect either drug to interfere with the metabolism of the other as they are metabolized by different cytochrome P450 enzymes.

All volunteers will receive at minimum daily phone calls during the 2 weeks prior to endotoxin instillation to verify full compliance with the outpatient pioglitazone and zileuton treatments. Video conferences to document compliance will be performed whenever possible. If any evidence of noncompliance with the outpatient drug regimen is discovered before inpatient study procedures begin (which will include inability to contact the volunteer during the outpatient treatment period for compliance verification), the volunteer will be withdrawn from the study.

- - 1. Drug Preparation

Pioglitazone and zileuton will be obtained through commercial sources. Pioglitazone will be overencapsulated with a size 00 capsule (Gallipot, Inc., St. Paul, MN) for blinding and cushioned with lactose monohydrate NF (Spectrum Co., Gardena, CA). Zileuton tablets will be crushed and then overencapsulated with a size 000 gelatin capsule (Gallipot, Inc., St. Paul, MN) for blinding. Matching placebo pills containing lactose for both pioglitazone and zileuton will also be created. All encapsulation and the distribution of pills into a medication container will be performed by the Investigational Drug Service of the Barnes-Jewish Hospital Pharmacy, which has supported our two prior clinical trials. Endotoxin will be prepared and administered as previously described.

- - 1. Medication Diary

Volunteers will be asked to document when they take the pioglitazone/placebo and zileuton/placebo pills on a spreadsheet (Appendix III). They will be asked to write the time that they took the pills required in each box on the spreadsheet.

- 1. **Bronchoscopic Administration of Endotoxin and Bronchoalveolar Lavage**
     1. Bronchoscopy and endotoxin administration

The volunteer will be given 2 doses of either trimethoprim/sulfamethoxazole or amoxicillin, approximately 12 hours apart, prior to bronchoscopy. The volunteer will be continuously monitored by heart rhythm, oxygen saturation, and non-invasive blood pressure monitoring and will receive pre-medications for conscious sedation according to standard protocols through the Barnes-Jewish Hospital Bronchoscopy Suite (Appendix I). A 5F balloon-tipped monitoring catheter will be inserted via a fiberoptic bronchoscope to occlude a single lung segment (usually the lateral segment of the right middle lobe). Endotoxin (4 ng/kg, in 2 ml water) will be administered through the distal port of the catheter. The catheter will be flushed with 10 ml of normal saline, followed by 10 ml air. The catheter will be kept in place for 30 sec, after which the balloon will be deflated. The catheter and bronchoscope will then be withdrawn and the head of the bed raised to 30^o^. Post-procedure monitoring, including frequent vital signs and oxygen saturation monitoring (q 30 min x 4 hr, q 1 hr x 4 hr, and then q 4 hr until completion), will take place in the CRU.

- - 1. Drug preparation

The endotoxin (E. coli O:113, Reference Endotoxin) will be reconstituted with sterile water to a final concentration of 2,000 endotoxin units/ml. The dose of 4 ng/kg will be prepared to a final volume of 2 ml. Dr. Delphine Chen is the current Sponsor-Investigator for IND #100042 for endotoxin administration to humans.

- - 1. Bronchoalveolar lavage (BAL)

After wedging the bronchoscope into the same segmental airway used for endotoxin administration, three sequential 50 ml volumes of sterile warmed (37°C) normal saline will be instilled through the suction channel of the bronchoscope. BAL fluid will be recovered by gentle aspiration after each aliquot, pooled, and kept on ice. Cells obtained by BAL will be separated from the fluid immediately, fixed, and stained with Wright-Giemsa stain for determining cell counts and assayed for [^3^H]deoxyglucose ([^3^H]DG) uptake. The BAL fluid itself will be frozen and stored for later cytokine analysis.

- 1. **PET-CT Imaging with [^18^F]FDG**
     1. Imaging acquisition protocols:

Imaging studies will be performed on 10300 West Pavilion of Barnes-Jewish Hospital in the Center for Clinical Imaging Research (CCIR). Imaging data will be acquired on a Siemens Biograph 40 TruePoint Tomography PET/CT scanner and will be reconstructed using filtered back projection. Research participants will be fitted with a pneumatic bellows device that wraps around the patient’s abdomen and positioned supine in the scanner. A low-dose CT scan (30 effective mAs) will be obtained with placement of the participant such that the right middle lobe is imaged. The CT scan will be obtained at normal end-expiration, as several studies have demonstrated that this protocol minimizes misregistration artifact. After completing the transmission scan, 10 mCi of [^18^F]FDG will be injected i.v. at the start of a 60-minute dynamic scan acquisition and the i.v. catheter flushed with 10 ml saline. Imaging will be obtained with the following framing schedule: 24 5–sec, 6 3-minute and 8 5-minute frames. The pneumatic bellows device simply records movement of the abdomen to obtain respiratory phase information during the PET scan for image processing after completion of the PET imaging acquisition. Blood samples will also be collected during this time to determine the “input function.” A second end-inspiration, low-dose CT scan (50 effective mAs) will also be obtained at the end of the PET/CT acquisition. Additional information is located in Appendix II.

- 1. **Blood Collection**

In addition to the screening procedures as described in Study Procedures for Specific Aim 1, an additional 15 ml of blood will be collected at the screening visit, when the volunteer is admitted to the Clinical Research Unit (CRU, formerly GCRC), and again at the time of the second PET scan. White blood cells will be isolated from these samples for determination of [^3^H]DG uptake (since we do not expect neutrophil activation peripherally, these will serve as a negative control for [^3^H]DG uptake in the cells collected by BAL). We will also assay for CD11b expression in the cells (specifically in the neutrophils) using flow cytometry. A portion of these blood samples will also be sent for standard blood counts and lab chemistry, including C-reactive protein determination, to confirm that no systemic inflammation is induced by the endotoxin. The levels of the different leukotrienes and other levels of cytokines that are impacted by the drug treatments (which includes several of the secondary endpoints) will be tested by ELISA in the blood obtained at these time points. These markers will serve as internal negative controls for the same assays to be performed on the BAL samples.

- 1. **Videotaping of Bronchoscopy Procedures**

Videotapes of the airways only will be obtained during each bronchoscopy procedure. There will be no pictures of facial or other features that might provide the potential for breach of confidentiality. The videos will be identified by code numbers only; the master list will be kept in a location separate from the videotapes. While the study is active, videotapes will be stored in a secured location in the investigator’s office. Only research team members will have access to these videotapes. These videotapes will be stored indefinitely but all identifying source documents will be destroyed 5 years after the research is completed.

- 1. **Specific Procedures Timeline**

**Outpatient Screening Visit:**

1. Informed consent will be reviewed with and obtained from participant (Preview of consent to be performed by telephone prior to screening visit. Participant will also receive a copy of the consent by email or mail prior to screening visit).
2. History and physical will be performed.
3. Screening blood work (CBC, CMP, PT/PTT, CRP & ESR), a urinalysis, and pulmonary function tests, chest x-ray, and electrocardiogram will be obtained. A serum pregnancy test will also be obtained, if applicable. Some of the blood collected will be tested with research lab assays to confirm the efficacy of the study drugs. A urine sample will also be obtained for research lab assays for this same purpose.
4. If the volunteer passes all screening tests, s/he will be enrolled into the following groups (in order): pioglitazone plus oral placebo, zileuton plus oral placebo, pioglitazone plus zileuton, or two oral placebos.

**Outpatient Drug Treatment:**

1. Each volunteer will take the following:
   1. Pioglitazone 45 mg or matching placebo by mouth once dailyfor 2 weeks prior to admission.
   2. Zileuton 600 mg or matching placebo by mouth with food qid for 5 days prior to admission.

**Day 1:**

1. The volunteer will be admitted to the CRU.
2. Two (2) intravenous catheters will be started, one in each arm. Both catheters will be saline locked or maintained as continuous i.v. with normal saline at a keep-vein-open drip rate (10-20cc/hr).
3. Blood will be drawn for baseline testing, including screening labs and specific research assays for this study. A portion of this blood will also be tested for the TLR4 polymorphism (exploratory endpoints). A small drop of blood will also be taken to check a blood glucose level. A urine sample will also be obtained for research lab assays.
4. A urine pregnancy test will also be obtained, if applicable, to confirm non-pregnancy prior to [^18^F]FDG administration.
5. FDG-PET/CT scan will be obtained followed immediately by a second CT scan*.* The volunteer will have been instructed to be NPO for at least 4 hours prior to the PET scan.
6. Twelve hours before the bronchoscopy is scheduled, the subject will receive one tablet of oral sulfamethoxazole-trimethoprim (80/400) or oral amoxicillin (500 mg.) by mouth.
7. The volunteer will be NPO after midnight in preparation for bronchoscopy the following morning.
8. Pioglitazone/placebo will be given once daily and zileuton/placebo will be given qid until the morning of Day 3.

**Day 2:**

1. The volunteer’s temperature will be checked twice during the hour before their bronchoscopy.
2. One hour prior to bronchoscopy, the volunteer will receive a second dose of one tablet of the oral antibiotic: sulfamethoxazole/trimethoprim (80/400) or amoxicillin (500 mg.).
3. The volunteer may receive pre-procedure medications (Demerol, Vistaril), before the bronchoscopy.
4. During the bronchoscopy, endotoxin 4 ng/kg will be instilled into a middle lobe segment of the right lung as described above. The volunteer’s vital signs and oxygen saturation will be monitored closely. Oxygen will be administered to maintain their saturation level per protocol.
5. Once the volunteer returns to his/her room at the CRU, s/he will be observed per protocol for the rest of the day. These observations include: checks of vital signs, oxygenation, and symptoms (q 30 min x 4 hr, q 1 hr x 4 hr, and then q 4 hr until completion).
6. After the bronchoscopy, the i.v. fluids will be maintained until the volunteer can tolerate oral medications and nutrition.
7. Acetaminophen will be administered as needed.
8. The volunteer will be NPO after midnight in preparation for the PET scan and BAL the next day.
9. I.v. fluids will be given beginning at midnight to maintain hydration while volunteer is NPO.

**Day 3:**

1. The subject will have blood drawn in the morning for repeat testing of CBC, CMP, CRP and ESR. Additional blood will again be drawn for research lab assays. Any values outside normal expected values (see exclusion criteria) will be reviewed by one of the investigators, and a decision will be made concerning whether continued observation in the hospital is required or out-patient follow-up is needed until resolution. A urine sample will again be obtained for the same research lab assays as above.
2. If the second i.v. placed on Day 1 is not still functioning, a second i.v. catheter will be placed in the subject’s arm for the PET study.
3. The last dose of pioglitazone/placebo and zileuton/placebo will be administered in the morning.
4. A small drop of blood will be taken to check a blood glucose level.
5. FDG-PET/CT and second CT scan will be obtained in exactly the same manner as was done on Day 1 of this study.
6. Pulmonary function tests will be obtained.
7. The volunteer will be escorted back to his/her room at the CRU and will begin preparation for the 2^nd^ bronchoscopy, which is for the bronchoalveolar lavage.
8. The subject may receive “pre-op” medications (Demerol, Vistaril) before the bronchoscopy as well as medication during the bronchoscopy (Versed and fentanyl). This will be at the discretion of the study physician to reduce any discomfort during the bronchoscopy.
9. During the bronchoscopy, the subject’s vital signs and oxygen saturation will be monitored closely. Oxygen will be administered to maintain their saturation level per protocol.
10. After the bronchoscopy and bronchoalveolar lavage (BAL), the subject will be monitored closely for the next several hours. Intravenous fluids will be maintained until the patient can tolerate oral medications and nutrition. After 4 hours of monitoring, if he/she is clinically stable, the monitors will be removed and s/he will be allowed to eat. Once fully recovered from the medications, the study physician will determine if the volunteer meets the criteria to be discharged to go home or whether s/he should remain in the CRU for overnight observation.

During the study, the volunteer will have PFTs measured after endotoxin instillation but prior to the BAL. BAL will not be performed if the FEV_1_ has decreased >20% from the pre-endotoxin baseline value. The change in pulmonary function will be followed until resolved or stabilized.

All volunteers will be observed as an inpatient for a minimum of 24 hours after endotoxin instillation (including any volunteers who receive endotoxin but withdraw from the study for any reason). The study will be terminated for any volunteer who experiences a serious adverse event (defined below). A follow-up call will be made 24 hours and 7+2 days after discharge to assess for any health problems.

- 1. **Criteria for discharge from the CRU**

Subjects must meet the following criteria to be eligible for discharge the day after endotoxin instillation:

- No respiratory symptoms (cough, difficulty breathing, chest pain, wheezing, or excessive secretions) or need for intervention or assistance with O_2_ therapy or bronchodilators.
- Oral temperature less than or equal to 38.0° C.
- Heart rate 60-100 beats per minute.
- Systolic blood pressure >80 mmHg, <20% increase from baseline, <20% decrease from baseline.
  - - Diastolic blood pressure <20% increase from baseline, <20% decrease from baseline.
  - Pulse oximetry reading SpO_2_>90% without the use of supplemental O_2_ therapy.
  - No intervention required for treatment of malaise, or myalgia.
  - FEV_1_ at least 81% of baseline.
  - Absence of any other symptom or set of symptoms which in the judgment of the investigator requires additional observation in the CRU or hospital.

Beyond the first day after endotoxin instillation, discharge decision will be based on the investigator’s best medical judgment using the following criteria: lack of progression of the abnormality necessitating continued observation, stabilization or clinical improvement of the abnormality, and whether there is need for ongoing treatment.

**Post-treatment period (defined as time of discharge from CRU until 10 days post-discharge).**

The volunteer will be given contact numbers for any problems encountered during this follow-up period. The research staff will contact the volunteer the next day (Day 4) and 7-9 days post discharge concerning any health-related problems.

1. **ADVERSE EVENT REPORTING AND DATA/SAFETY MONITORING**
   1. **Definitions**

The following definitions of adverse event and serious adverse event will be used for this study.

**Adverse event (AE):** AEs are defined conventionally, as any untoward medical occurrence during the planned observation period in a research participant that develops following test drug administration. The AE and drug administration do not have to be causally related.

**Serious adverse event (SAE):** SAEs are also defined conventionally as any medical occurrence that results in death, is life-threatening, requires inpatient hospitalization, results in persistent or significant disability, is a congenital anomaly or birth defect, or is an event requiring medical intervention to prevent any of these examples of an SAE.

SAEs may be mild (transient, easily tolerated by the participant), moderate (causes discomfort or interrupts the study or the participant’s usual activities), or severe (causes considerable interference with usual activities) in severity. Any criterion outside of the above boundaries must be documented as present for >2-4 hours, in order to be considered ‘more severe’.

- 1. **Causality of adverse events**

The causality of each AE in terms of relationship to administration of test drug will be assessed as definite (reasonable temporal relationship, with or without supporting laboratory data), probable (reasonable temporal relationship and other possible causes can be reasonably excluded), possible (reasonable temporal relationship and other possible causes are at least as or more likely), and unrelated (temporal relationship is not reasonable or other causes are reasonably more likely).

## Safety Monitoring

We have not had any serious adverse events in the two clinical trials we have conducted, nor have any been reported in additional publications using this model[^38-40^](#_ENREF_38) since the original report was published[^35^](#_ENREF_35). Vital sign monitoring, inpatient observation, and outpatient follow-up (calls at 1 day and at 7+2 days after discharge) will be performed as we have published previously. The study will be terminated for any volunteer who experiences a SAE as defined above.

An independent, internal DSMB will monitor this trial. The DSMB will include senior investigators with expertise in critical care and infectious diseases and a statistician. The DSMB will make recommendations to ensure data integrity and the safety of the volunteers.

DSMB Activities:

1) Review the research protocol and approve plans for data and safety monitoring.

2) Evaluate the progress of the study, including periodic assessments of data quality and timeliness, participant recruitment, accrual and retention, participant risk versus benefit, and other factors that can affect study outcome. Monitoring will also consider factors external to the study when interpreting the data, such as scientific or therapeutic developments that may have an impact on the safety of the participants or the ethics of the study. The principal investigator will make available to the DSMB any newly published information related to the use of endotoxin, pioglitazone, or zileuton which may affect patient safety.

3) Make recommendations to Washington University School of Medicine, the IRB, and investigators concerning continuation or conclusion of the trial.

4) Submit an annual report to the IRB.

5) Protect the confidentiality of the trial data and the results of monitoring.

6) Review data and safety issues (including any and all adverse events) and determine, with information provided by the study staff, whether the events should be regarded as study-related or unrelated. The DSMB will be empowered to stop the study, at their discretion, for safety reasons.

DSMB meeting frequency: The DSMB will meet to review the above listed information after the first two patients. They will then decide whether the study can go forward as planned, whether modifications are to be made in any procedures, and when to review the next set of data (but not less than every 8 patients).

Stopping rules: The study will be stopped *for any individual subject* in the case of any SAE, defined prospectively. New recruitment/enrollment will be discontinued after any SAE until satisfactorily reviewed by the DSMB. The DSMB will make recommendations, if any, for changes to recruitment practices, the informed consent document, or safety/monitoring practices. All such recommendations will be communicated to the IRB, RDRC, and CRU.

The FDA will be notified of any study stoppages. Recruitment for study participation will resume *only* when we have received clearance from the FDA to do so. The entire study will be discontinued completely in the event of any study-related life-threatening event or death.

- 1. **Collecting/reporting of adverse events**

In the study in normal volunteers, all adverse experiences, whether expected or unexpected, will be recorded in the case report form (see Appendix III for samples) and reported to the DSMB for this study. Any adverse experience that occurs to a greater severity than expected will be reported to the IRB, RDRC, CRU, and FDA.

The IRB (Washington University School of Medicine Human Research Protection Office), RDRC, and CRU will be notified of any SAE within 10 working days of occurrence. The FDA will be sent an IND Safety Report within 10 working days of occurrence of any SAE. If the event is fatal or life threatening, the DSMB, IRB, and RDRC will be notified of the event within twenty-four hours. The CRU and FDA will be notified by telephone no later than 3 days after occurrence.

The IRB, RDRC, CRU, and FDA will receive annual reports regarding all adverse experiences.

- 1. **Follow-up of serious adverse events**

All SAEs will be followed up until resolution or permanent outcome of the event. All follow-up information will be included in the case report form.

## Potential Risks

### 5.6.1. Risks associated with bronchoscopy and bronchoalveolar lavage

General:

Potential risks are related to both the administration of the medications necessary to perform the procedures (discussed later), plus those related to the actual procedure of bronchoscopy itself. The risks associated with the procedure are:

*Likely:* Coughing (at times severe) is the most common side effect of bronchoscopy. Hoarseness, loss of voice and sore throat are often experienced temporarily after bronchoscopy.

*Rare:* These include allergic reactions to drugs administered, low blood oxygen, increased heart rate, palpitations, irregular heart rhythm, pneumonia, fever, rigors, abnormalities in chest X-rays, difficulty breathing, respiratory failure, airway spasm and narrowing, trauma to the nose, airway, or lung, or death.

### 5.6.2. Risks associated with bronchoalveolar lavage

*Likely:* BAL usually causes a slight decrease in oxygen levels in the blood for up to a couple of hours after BAL. This may affect breathing and exercise capacity.

*Less Likely:* Post-BAL fever, sudden onset of stiffness, and a vague feeling of bodily discomfort has been reported in 10%-30% of patients 4-6 hours after the procedure. This usually resolves within 24 hours.

### 5.6.3. Risks associated with medications/drugs

**Endotoxin (intrabronchial instillation, 4 ng/kg):**

*Likely:* Mild to moderate respiratory symptoms which may include cough, difficulty breathing, chest pain, wheezing, or excessive secretions. These are expected to last less than 48 hours. Other side effects are also expected to last less than 24 hours. These include: mild fever, mild decrease in heart rate and blood pressure, a slight decreased ability to absorb oxygen into the body, and mild or moderate head-ache, muscle aches, and a vague feeling of bodily discomfort.

*Rare:* Endotoxin in the blood stream at high levels can cause serious reactions such as shock and death. Endotoxin in the lungs at high levels can cause reactions similar to severe pneumonia, leading to low oxygen levels, need for breathing machine support, heart attacks, brain damage, and death. However, there have been no deaths reported when endotoxin has been used in the manner described here for research purposes. There have also been no deaths reported when endotoxin has been administered intravenously or to the lungs in a mist during approved research protocols. Delphine L. Chen, M.D. is the Sponsor-Investigator for the IND (#100042) from the FDA for the bronchoscopic instillation of endotoxin (E. coli O:113, NIH Clinical Center Reference Endotoxin [CCRE]) into a lung segment of healthy volunteers. An amendment will be filed to add the protocols required for this proposal to the IND.

**Antibiotics used to decrease bacteria in the lung** (each normal volunteer will only receive one type, 1 dose 12 hours prior to and a second dose immediately prior to bronchoscopy):

Oral sulfamethoxazole*/*trimethoprim (80/400):

*Less Likely:* Allergic skin reactions to this medication are common and include rash, itching and sensitivity to the sunlight. Many people experience nausea, vomiting, or loss of appetite. Some people have developed liver problems when taking this medication.

*Rare:* Some people have experienced mental and nervous system side effects, which have included confusion, depression, hallucinations, seizures, difficulty with coordination, and fever. There have been reports of blood abnormalities related to taking this medication. Rarely, people have developed a severe allergic reaction (Steven-Johnson Syndrome) which included a severe rash, sores in their mouth, diarrhea, and inflammation of their digestive tract. This is more likely with longer use of the drug. Kidney inflammation resulting from taking this drug has been reported.

*This is a “sulfa” drug. As such, people with allergies to sulfa containing drugs may experience severe allergic reactions to it. In addition, people with G6PD deficiency can develop severe anemia from taking this drug. The side effects listed above are much less likely when the drug is taken only for one day, as in this protocol.

Oral amoxicillin:

*Less Likely:* Rash can occur in people who are allergic to this drug or who have an active infection with mononucleosis. Some people have developed diarrhea from taking this medication. Occasionally this drug has decreased a person’s ability to fight certain types of bacteria, resulting in a severe infection. There have been reports of mental and nervous system side effects, which have included seizures and fevers.

*These side effects are much less likely when the drug is taken only for one day, as in this protocol.

**Medications used prior to bronchoscopy** (each subject may receive one or more of these):

These medications are used according to standard policy and procedures for the performance of bronchoscopy at Barnes-Jewish Hospital. Research participants may receive Meperidine HCl (Demerol), Hydroxyzine (Vistaril), and Inhaled Lidocaine, 1% and 2%:

*Likely:* Common side effects include: dry nose, mouth, or throat, low blood pressure, lightheadedness, dizziness, drowsiness, constipation, nausea, vomiting, or weakness.

*Less Likely:* Occasionally people experience difficulty swallowing, sore throat, headache, stomach cramps, hunger, diarrhea, abdominal pain, nervousness, confusion, fatigue, difficulty urinating, difficulty breathing, pain at injection site, joint aches, or tremor.

*Rare***:** Rare reactions that have been reported include racing heartbeat, irregular heartbeat, eye pain, blurred vision, sensitivity to light, water retention, rash, angioedema (swelling around eyes and throat), numbness, cough, bloody nose, shock, mental depression, excitement, sedation, coma, agitation, hallucinations, or seizures.

**Medications used during bronchoscopy for sedation** (each participant may receive one or more of these): Midazolam HCl (Versed) and Fentanyl:

*Likely:* Common side effects include pain at injection site, hiccups, low blood pressure, slow heartbeat, drowsiness, nausea, vomiting, constipation, or slower breathing.

*Less Likely:* There is a slight chance that participants may experience headache, cough, blurred vision, dizziness, lightheadedness, abdominal cramps, difficulty breathing, excitement, confusion, loss of memory, irregular heartbeat, or their heart could stop beating.

*Rare:* Rare reactions that have been reported include convulsions, rash, wheezing, cold and clammy skin, difficulty urinating, rapid heartbeat, or mental confusion.

**Test drugs.** The following drugs are commercially available and FDA-approved. They are designated “test” drugs in the context of this research study.

Pioglitazone:

Adverse reactions reported for pioglitazone are based on chronic administration (at least 16 weeks, typically 26 weeks or longer) in patients with diabetes. It is expected that these reactions will be less likely with the 14-day total treatment protocol required for this study..

*Less Likely:* Upper respiratory tract infection, headache, sinusitis, myalgia, pharyngitis.

*Rare:* Hypoglycemia, heart failure, edema, pain in extremity, back or chest(these were seen with chronic administration of the drug in patients with diabetes; these are expected to be highly unlikely in healthy volunteers receiving this drug for 2 weeks). An increased risk of bladder cancer has been observed in patients taking this drug for at least 12 months or longer.

Zileuton:

Adverse reactions reported with zileuton are based on chronic administration (12-week treatment or longer). It is expected that these reactions will be less likely with the 5-day total treatment protocol required for this study. Small studies with treatment periods similar to the one used in this protocol have not reported adverse effects related to zileuton treatment.

*Less Likely:* Sinusitis, nausea, nasopharyngeal pain, gastrointestinal disorders (upper abdominal pain, diarrhea, dyspepsia, vomiting), rash, hypersensitivity, hepatotoxicity, headache, upper respiratory tract infection, low white blood cell counts.

*Rare:* Severe hepatic injury resulting in death, life-threatening liver injury with recovery, symptomatic jaundice, hyperbilirubinemia, and elevations of ALT over 8x upper limits of normal (case reports).

### 5.6.4. Risks associated with PET/CT imaging with [^18^F]FDG

Scan itself:

*Less likely:* Some people may find lying flat or wearing the pneumatic bellows device to be uncomfortable and experience shoulder or back discomfort as a result. The participant will need to lie flat for ~1½ hours for the PET/CT scan. If the pneumatic bellows device is the reason for discomfort, it will be removed. If the subject cannot tolerate lying flat at all, the study will be discontinued.

Exposure to radiation:

*Likely:* The amount of radiation exposure each person will receive from a 10 mCi injection of [^18^F]FDG plus the attenuation-correction CT scan and a second end-inspiration CT scan over the lungs for a single PET/CT session is equivalent to a uniform whole-body exposure of 0.55 rems. With the second low-dose CT scan, the total dose for 2 PET/CT plus 2 CT scans to complete the PET/CT imaging protocol for this study (total 2.0 rems), this is equivalent to ~40% of the allowable annual dose for radiation workers.

*The risk from radiation exposure of this size is too small to be measured directly. Participants will be provided with a “Radiation Fact Sheet” supplied by the Washington University School of Medicine, Division of Nuclear Medicine.

Radioactive Drugs:

[^18^F]FDG is an FDA-approved drug that is given in such small (“trace”) amounts that they have no known harmful effect. PET/CT scans with this tracer are performed routinely for clinical indications with no associated adverse effects reported in the history of its use.

It was recently reported by the FDA that the CT scans may cause the malfunction of electronic medical devices. However, volunteers with such implanted devices will be excluded from participating in this study.

### 5.6.5. Risks associated with blood draws, intravenous catheter insertion, non-invasive tests

Chest radiograph

*Likely:* The volunteer will be exposed to a small amount of radiation (0.01 rem), which is 0.2% of the allowable annual dose for radiation workers.

*The risk from radiation exposure of this size is too small to be measured directly. Participants will be provided with a “Radiation Fact Sheet” supplied by the Washington University School of Medicine, Division of Nuclear Medicine.

Blood draws and i.v. catheter insertion:

*Less Likely:* The insertion of a needle into a vein sometimes causes discomfort, bruising, or bleeding at the site of needle insertion. Occasionally some people experience dizziness or feel faint when a needle is placed in their arm.

*Rare:* The insertion of a needle into a vein rarely causes inflammation or infection.

Spirometry:

*Less Likely*: The effort required for a good spirometry test may cause shortness of breath. However, participants will be allowed to take rest breaks between measures if needed. Occasionally, during spirometry, people have experienced dizziness, wheezing, chest pain, racing or pounding heart, an upset stomach, severe shortness of breath, or coughing.

*Rare:* Rarely, people have fainted during spirometry. The test will not be continued in such a case.

Pregnancy:

The medications used for the bronchoscopy and the radiation exposure can cause birth defects and mental retardation to a fetus. All women volunteers for this study will have had a negative pregnancy test prior to further participation.

### 5.6.6. Risks associated with coded data

*Rare:* Each participant enrolled will have a consecutive patient ID number under which data will be coded. In addition, the following information may be used in association with coded data: initials and date of birth. There is a remote possibility of a breach of confidentiality. De-identified data will be stored indefinitely. Identifiable data will be destroyed 5 years after completion of the protocol.

## Adequacy of Protection Against Risks

- - 1. **Recruitment and Consent**

We will not enroll volunteers who work within our department. We will not schedule admission to the CRU until the volunteer has had a chance to read the entire consent and allowed as much time as they require to consider participating (there is no time pressure in which the volunteer must make their decision). We have added additional reminders regarding human research participants’ rights at the beginning of the informed consent.

Since this is an elective study, all participants will be given a written copy of the consent to read and discuss with the investigator or study coordinator more than 24 hours prior to making a decision about their participation. Volunteers will be allowed all the time they want to think about, and discuss, their decision to participate.

Initial discussion of the study with the trained research coordinator working with the principal investigator will occur at the WUSM Volunteer for Health office, the Center for Clinical Studies, or over the telephone. The investigator, co-investigator, or a research fellow (trained in the protection of human subjects for this study protocol) will review the consent with participants in the CRU at the time of their admission. All personnel have received instruction on the Responsible Conduct of Research.

- - 1. **Protections Against Risk**

We have chosen sequential enrollment into the study drug treatment groups so that we can effectively monitor for serious adverse events possibly associated with each drug treatment.

All reasonable measures will be taken to protect the confidentiality of clinical records and research data. The identity of the participants will not be revealed in any publication that might result from this research. Individuals involved in conducting this research project will have access to material collected from these research participants. The confidentiality of all study related records will be maintained according with State and Federal laws.

Results of interventions and evaluations of participating volunteers will be recorded in a clinical source document. These records will be stored in files locked within the investigator’s office and on local WUSM office computers with restricted access, and in the CRU medical records room, which also has access restricted to trained health care professionals.

Initial inclusion/exclusion criteria, physical exam, chest x-ray, ECG, spirometry, and blood tests are to assure us that we only enroll healthy volunteers with intact pulmonary function.

All necessary and appropriate procedures will be taken to prevent and monitor for the early detection of bronchoscopy-related complications. Continuous intravenous vascular access, supplemental oxygen, ECG, and pulse oximetry monitoring will be maintained during the bronchoscopy and for a minimum of 2 hours post bronchoscopy. BAL will be limited to one lobar segment or subsegment, and limited to three 50 ml instillations. Biopsies will not be performed.

Endotoxin has been given many times to healthy research volunteers by injection into a vein, or by inhalation of a mist of endotoxin. Recently, an NIH-sponsored study showed that instillation of endotoxin into a single small section of the lung resulted in an area of lung inflammation that can be triggered with doses 10-30 times smaller than those typically used when endotoxin has been inhaled as a mist.[^35^](#_ENREF_35) Another advantage of bronchial instillation of endotoxin is that rather than challenging the whole lung with endotoxin, the majority of the lung tissue is not affected by the endotoxin. Nick et al, investigators from another institution other than the NIH, recently reported a study with this same protocol without serious or unexpected adverse events.[^39^](#_ENREF_39) We also have reported 2 studies including 36 subjects in total without any serious adverse events.

In order to minimize lower airway bacterial contamination during bronchoscopy, volunteers will receive one tablet of oral sulfamethoxazole/trimethoprim (80/400) or one tablet of oral amoxicillin (500 mg.) 12 hours before the bronchoscopy is scheduled, and again immediately before the procedure. Participants will be NPO beginning at midnight to reduce the risk of aspiration during the bronchoscopy.

Extensive procedures have been set up and approved by our IRB and the FDA for monitoring of the participants during and after all procedures. Specific criteria have been established for discharge from the CRU and for follow-up after discharge. These criteria include specific definitions of adverse and serious adverse events, as described above.

Volunteers and their research specimens will be de-identified for the purpose of data collection into case report forms for analysis, using a letter designating the study and a numbering system to designate enrollment number. PET studies are coded sequentially in the order in which they have been completed. Individuals will have an ID number, as well as a separate PET study number for each PET study completed. Data and clinical records will be stored securely, locked in the investigator’s office or the medical records room at the CRU. Deidentified data, including videotapes obtained of the airways during bronchoscopy, will be stored indefinitely, but all identifying source documents will be destroyed 5 years after the research is completed.

All efforts will be made to reduce the dose of radiation that research volunteers are exposed to, including ongoing review of data to determine the minimum dose of radioactive tracer and minimum requirements for an adequate CT scan. To minimize risk associated with radiation exposure, pregnant and lactating women and children 18 years old or younger are excluded from participation. In order to reduce radiation exposure, participants will be instructed to empty their bladders immediately after PET scanning is completed (about 70 minutes after tracer administration).

1. **DATA ANALYSIS/STATISTICAL METHODS**
   1. **Sample Size Calculation**

Our recently completed drug treatment study in healthy volunteers receiving intrabronchial endotoxin demonstrated an approximately 50% decrease with ~25% standard deviation in the *K*_i_ after treatment with lovastatin when compared to placebo (see Preliminary Data Section). A sample size of N=6 in each group will allow us to detect a similar magnitude of difference with approximately the same amount of variability in the measurement (23% standard deviation of residuals) with a power of 0.8 and alpha of 0.05.

- 1. **Statistical Analysis**

An interim analysis will be performed after the first treatment group is filled. If there is a minimum of a 30% drop in the post-endotoxin *K*_i_ from the baseline *K*_i_, enrollment will continue into the next 3 groups. If no change in *K*_i_ is observed at this interim analysis, we will continue to enroll volunteers into the zileuton+placebo and placebo+placebo groups but will not enroll volunteers into the pioglitazone+zileuton group. We recognize that this approach reduces the power of the study but felt this was justified as we would still be able to adequately test our hypotheses while minimizing the risks to the volunteers for this study. At the end of the study, a three-way mixed model analysis of variance (zileluton, pioglitazone, and time ANOVA) will be used to assess the effect of the drug treatments on change in *K*_i_ from baseline and *K*_i_, after treatment, the primary endpoints among the treatment groups. Appropriate prespecified contrasts will be used for the primary tests. An exploratory analysis using a two-way ANOVA will also be performed to analyze the treatment effects on the BAL total cell counts, BAL neutrophil counts, PPAR-γ transcriptional activity, LXA_4_, LTB_4_, IL-8, and TNF-α levels (by ELISA) in the BAL fluid, and 5-LO, 15-LO and PPAR-gamma mRNA levels in BAL cells, although this study will not be powered specifically to detect these relationships. Correlations between the post-endotoxin *K*_i_ and the BAL and urine variables will be performed using standard linear regression with calculation of the coefficient of determination (R^2^). Correlations will also be explored among serum and BAL measurements and *K*_i_. Any volunteer with incomplete scan data will not be included in the analysis. Any other missing data (i.e. missing BAL data point) will be handled by excluding that data point from that particular analysis. We will also assure that no bias was introduced by this handling of the missing data by using multiple imputation for all missing data.

Correlations will also be explored between serum measurements and both *K*_i_ and BAL measurements. CT density and airway thickness will also be correlated with *K*_i_ and BAL measurements. Respiratory-gated SUV will be correlated with SUV from non-gated PET data as an exploratory analysis to determine the potential value of respiratory-gating for this imaging application. The presence of TLR4 polymorphisms will be correlated with *K*_i_ and BAL measurements to explore whether these SNPs predict hypo-responsiveness to endotoxin as previously published[^28^](#_ENREF_28).

1. **STUDY ADMINISTRATION**

**7.1. Direct Access to Source Data and Documents**

The Principal Investigator, Co-Investigator, and Coordinators will have access to patient-identifying data. Image and data analysts will have access only to deidentified, coded data.

**7.2. Ethics**

**7.2.1. Institutional Review Board (IRB) and Regulatory Approvals**

The Principal Investigator will ensure that the protocol is approved by the IRB, RDRC, and FDA prior to commencing the trial. All amendments to the protocol will also be approved by each of these organizations prior to execution. The only circumstance in which an amendment may be initiated prior to IRB approval is when the change is necessary to eliminate apparent immediate hazards to the subjects. In that event, the investigator must notify the IRB, RDRC, and FDA in writing within 5 working days after the implementation.

### 7.2.2. Ethical Conduct of the Trial

The trial will be performed in accordance with the protocol, International Conference on Harmonization Good Clinical Practice guidelines, and applicable local regulatory requirements and laws.

### 7.2.3. Subject Information and Consent

All efforts will be made to assure subject confidentiality by using subject identification code numbers and initials that correspond to treatment data in the computer files.

Written informed consent will be obtained from each participant prior to any study related procedures being performed.  A copy of the signed and dated consent document will be given to each subject.  The original signed and dated informed consent document must be maintained in the study files at the investigative site and be available for Sponsor or designee review.  The informed consent document must meet all IRB/EC and regulatory requirements.

## Records Retention and Data Archival

Deidentified data will be stored indefinitely. Records containing patient-identifying data will be stored per HIPAA regulations for 5 years after the close of the study and then destroyed. Deidentified videotapes will be stored indefinitely.

# REFERENCES

1. Lee, W.L. and G.P. Downey, *Neutrophil activation and acute lung injury.* Curr Opin Crit Care, 2001. **7**(1): p. 1-7.

2. Martin, T.R., *Neutrophils and lung injury: getting it right.* J Clin Invest, 2002. **110**(11): p. 1603-5.

3. Kruger, P., K. Fitzsimmons, D. Cook, M. Jones, and G. Nimmo, *Statin therapy is associated with fewer deaths in patients with bacteraemia.* Intensive Care Med, 2006. **32**(1): p. 75-9.

4. Davis, P.B., *Cystic fibrosis since 1938.* Am J Respir Crit Care Med, 2006. **173**(5): p. 475-82.

5. Konstan, M.W., K.A. Hilliard, T.M. Norvell, and M. Berger, *Bronchoalveolar lavage findings in cystic fibrosis patients with stable, clinically mild lung disease suggest ongoing infection and inflammation.* Am J Respir Crit Care Med, 1994. **150**(2): p. 448-54.

6. Chung, K.F., *Inflammatory mediators in chronic obstructive pulmonary disease.* Curr Drug Targets Inflamm Allergy, 2005. **4**(6): p. 619-25.

7. Barnes, P.J., *Immunology of asthma and chronic obstructive pulmonary disease.* Nat Rev Immunol, 2008. **8**(3): p. 183-92.

8. Fujimoto, K., M. Yasuo, K. Urushibata, M. Hanaoka, T. Koizumi, and K. Kubo, *Airway inflammation during stable and acutely exacerbated chronic obstructive pulmonary disease.* Eur Respir J, 2005. **25**(4): p. 640-6.

9. Hogg, J.C., F. Chu, S. Utokaparch, R. Woods, W.M. Elliott, L. Buzatu, R.M. Cherniack, R.M. Rogers, F.C. Sciurba, H.O. Coxson, and P.D. Pare, *The nature of small-airway obstruction in chronic obstructive pulmonary disease.* N Engl J Med, 2004. **350**(26): p. 2645-53.

10. Jatakanon, A., C. Uasuf, W. Maziak, S. Lim, K.F. Chung, and P.J. Barnes, *Neutrophilic inflammation in severe persistent asthma.* Am J Respir Crit Care Med, 1999. **160**(5 Pt 1): p. 1532-9.

11. Lamblin, C., P. Gosset, I. Tillie-Leblond, F. Saulnier, C.H. Marquette, B. Wallaert, and A.B. Tonnel, *Bronchial neutrophilia in patients with noninfectious status asthmaticus.* Am J Respir Crit Care Med, 1998. **157**(2): p. 394-402.

12. Sur, S., T.B. Crotty, G.M. Kephart, B.A. Hyma, T.V. Colby, C.E. Reed, L.W. Hunt, and G.J. Gleich, *Sudden-onset fatal asthma. A distinct entity with few eosinophils and relatively more neutrophils in the airway submucosa?* Am Rev Respir Dis, 1993. **148**(3): p. 713-9.

13. Riise, G.C., B.A. Andersson, C. Kjellstrom, G. Martensson, F.N. Nilsson, W. Ryd, and H. Schersten, *Persistent high BAL fluid granulocyte activation marker levels as early indicators of bronchiolitis obliterans after lung transplant.* Eur Respir J, 1999. **14**(5): p. 1123-30.

14. Riise, G.C., A. Williams, C. Kjellstrom, H. Schersten, B.A. Andersson, and F.J. Kelly, *Bronchiolitis obliterans syndrome in lung transplant recipients is associated with increased neutrophil activity and decreased antioxidant status in the lung.* Eur Respir J, 1998. **12**(1): p. 82-8.

15. Barnes, P.J. and B.R. Celli, *Systemic manifestations and comorbidities of COPD.* Eur Respir J, 2009. **33**(5): p. 1165-85.

16. Koehler, D.R., G.P. Downey, N.B. Sweezey, A.K. Tanswell, and J. Hu, *Lung inflammation as a therapeutic target in cystic fibrosis.* Am J Respir Cell Mol Biol, 2004. **31**(4): p. 377-81.

17. Nichols, D.P., M.W. Konstan, and J.F. Chmiel, *Anti-inflammatory therapies for cystic fibrosis-related lung disease.* Clin Rev Allergy Immunol, 2008. **35**(3): p. 135-53.

18. Chen, D.L. and D.P. Schuster, *Imaging pulmonary inflammation with positron emission tomography: a biomarker for drug development.* Mol Pharm, 2006. **3**(5): p. 488-95.

19. Taylor, I.K., A.A. Hill, M. Hayes, C.G. Rhodes, K.M. O'Shaughnessy, B.J. O'Connor, H.A. Jones, J.M. Hughes, T. Jones, N.B. Pride, and R.W. Fuller, *Imaging allergen-invoked airway inflammation in atopic asthma with [18F]-fluorodeoxyglucose and positron emission tomography.* Lancet, 1996. **347**(9006): p. 937-40.

20. Jones, H., S. Sriskandan, A. Peters, N. Pride, T. Krausz, A. Boobis, and C. Haslett, *Dissociation of neutrophil emigration and metabolic activity in lobar pneumonia and bronchiectasis.* Eur Respir J, 1997. **10**(4): p. 795-803.

21. Jones, H.A., P.S. Marino, B.H. Shakur, and N.W. Morrell, *In vivo assessment of lung inflammatory cell activity in patients with COPD and asthma.* Eur Respir J, 2003. **21**(4): p. 567-73.

22. Chen, D.L., T.W. Ferkol, M.A. Mintun, J.E. Pittman, D.B. Rosenbluth, and D.P. Schuster, *Quantifying pulmonary inflammation in cystic fibrosis with positron emission tomography.* Am J Respir Crit Care Med, 2006. **173**(12): p. 1363-9.

23. Klein, M., M. Cohen-Cymberknoh, S. Armoni, D. Shoseyov, R. Chisin, M. Orevi, N. Freedman, and E. Kerem, *18F-fluorodeoxyglucose-PET/CT imaging of lungs in patients with cystic fibrosis.* Chest, 2009. **136**(5): p. 1220-8.

24. Bush, A., *Treatment of cystic fibrosis: time for a new paradigm?* Chest, 2009. **136**(5): p. 1197-9.

25. Zhou, Z., J. Kozlowski, A.L. Goodrich, N. Markman, D.L. Chen, and D.P. Schuster, *Molecular imaging of lung glucose uptake after endotoxin in mice.* Am J Physiol Lung Cell Mol Physiol, 2005. **289**(5): p. L760-8.

26. Chen, D.L. and D.P. Schuster, *Positron emission tomography with [18F]fluorodeoxyglucose to evaluate neutrophil kinetics during acute lung injury.* Am J Physiol Lung Cell Mol Physiol, 2004. **286**(4): p. L834-40.

27. Chen, D.L., D.B. Rosenbluth, M.A. Mintun, and D.P. Schuster, *FDG-PET imaging of pulmonary inflammation in healthy volunteers after airway instillation of endotoxin.* J Appl Physiol, 2006. **100**(5): p. 1602-9.

28. Arbour, N.C., E. Lorenz, B.C. Schutte, J. Zabner, J.N. Kline, M. Jones, K. Frees, J.L. Watt, and D.A. Schwartz, *TLR4 mutations are associated with endotoxin hyporesponsiveness in humans.* Nat Genet, 2000. **25**(2): p. 187-91.

29. Mullarkey, M., J.R. Rose, J. Bristol, T. Kawata, A. Kimura, S. Kobayashi, M. Przetak, J. Chow, F. Gusovsky, W.J. Christ, and D.P. Rossignol, *Inhibition of endotoxin response by e5564, a novel Toll-like receptor 4-directed endotoxin antagonist.* J Pharmacol Exp Ther, 2003. **304**(3): p. 1093-102.

30. Suffredini, A.F., H.D. Hochstein, and F.G. McMahon, *Dose-related inflammatory effects of intravenous endotoxin in humans: evaluation of a new clinical lot of Escherichia coli O:113 endotoxin.* J Infect Dis, 1999. **179**(5): p. 1278-82.

31. van der Poll, T., E. Endert, S.M. Coyle, J.M. Agosti, and S.F. Lowry, *Neutralization of TNF does not influence endotoxininduced changes in thyroid hormone metabolism in humans.* Am J Physiol, 1999. **276**(2 Pt 2): p. R357-62.

32. Verbon, A., P.E. Dekkers, T. ten Hove, C.E. Hack, J.P. Pribble, T. Turner, S. Souza, T. Axtelle, F.J. Hoek, S.J. van Deventer, and T. van der Poll, *IC14, an anti-CD14 antibody, inhibits endotoxin-mediated symptoms and inflammatory responses in humans.* J Immunol, 2001. **166**(5): p. 3599-605.

33. Wilson, M., R. Blum, P. Dandona, and S. Mousa, *Effects in humans of intravenously administered endotoxin on soluble cell-adhesion molecule and inflammatory markers: a model of human diseases.* Clin Exp Pharmacol Physiol, 2001. **28**(5-6): p. 376-80.

34. Suffredini, A.F. and J.G. O'Grady, *Pathophysiologial responses to endotoxin in humans*. Endotoxin in health and disease, ed. O.S. Braude S, Vogel SN, Morrison DC. Vol. 1st ed. 1999, New York: Marcel Dekker. 817-30.

35. O'Grady, N.P., H.L. Preas, J. Pugin, C. Fiuza, M. Tropea, D. Reda, S.M. Banks, and A.F. Suffredini, *Local inflammatory responses following bronchial endotoxin instillation in humans.* Am J Respir Crit Care Med, 2001. **163**(7): p. 1591-8.

36. Abraham, E., J.A. Nick, T. Azam, S.H. Kim, J.P. Mira, D. Svetkauskaite, Q. He, M. Zamora, J. Murphy, J.S. Park, K. Overdier, and C.A. Dinarello, *Peripheral blood neutrophil activation patterns are associated with pulmonary inflammatory responses to lipopolysaccharide in humans.* J Immunol, 2006. **176**(12): p. 7753-60.

37. Chen, D.L., T.J. Bedient, J. Kozlowski, D.B. Rosenbluth, W. Isakow, T.W. Ferkol, B. Thomas, M.A. Mintun, D.P. Schuster, and M.J. Walter, *[18F]fluorodeoxyglucose positron emission tomography for lung antiinflammatory response evaluation.* Am J Respir Crit Care Med, 2009. **180**(6): p. 533-9.

38. Hohlfeld, J.M., K. Schoenfeld, M. Lavae-Mokhtari, F. Schaumann, M. Mueller, D. Bredenbroeker, N. Krug, and R. Hermann, *Roflumilast attenuates pulmonary inflammation upon segmental endotoxin challenge in healthy subjects: a randomized placebo-controlled trial.* Pulm Pharmacol Ther, 2008. **21**(4): p. 616-23.

39. Nick, J.A., C.D. Coldren, M.W. Geraci, K.R. Poch, B.W. Fouty, J. O'Brien, M. Gruber, S. Zarini, R.C. Murphy, K. Kuhn, D. Richter, K.R. Kast, and E. Abraham, *Recombinant human activated protein C reduces human endotoxin-induced pulmonary inflammation via inhibition of neutrophil chemotaxis.* Blood, 2004. **104**(13): p. 3878-85.

40. van der Poll, T., M. Levi, J.A. Nick, and E. Abraham, *Activated protein C inhibits local coagulation after intrapulmonary delivery of endotoxin in humans.* Am J Respir Crit Care Med, 2005. **171**(10): p. 1125-8.

41. de Torre, C., S.X. Ying, P.J. Munson, G.U. Meduri, and A.F. Suffredini, *Proteomic analysis of inflammatory biomarkers in bronchoalveolar lavage.* Proteomics, 2006. **6**(13): p. 3949-57.

42. Chen, D.L., M.A. Mintun, and D.P. Schuster, *Comparison of methods to quantitate 18F-FDG uptake with PET during experimental acute lung injury.* J Nucl Med, 2004. **45**(9): p. 1583-90.

43. Belvisi, M.G., D.J. Hele, and M.A. Birrell, *Peroxisome proliferator-activated receptor gamma agonists as therapy for chronic airway inflammation.* Eur J Pharmacol, 2006. **533**(1-3): p. 101-9.

44. Cuzzocrea, S., *Peroxisome proliferator-activated receptors and acute lung injury.* Curr Opin Pharmacol, 2006. **6**(3): p. 263-70.

45. Serhan, C.N. and P.R. Devchand, *Novel antiinflammatory targets for asthma. A role for PPARgamma?* Am J Respir Cell Mol Biol, 2001. **24**(6): p. 658-61.

46. Standiford, T.J., V.G. Keshamouni, and R.C. Reddy, *Peroxisome proliferator-activated receptor-{gamma} as a regulator of lung inflammation and repair.* Proc Am Thorac Soc, 2005. **2**(3): p. 226-31.

47. Ricote, M., A.C. Li, T.M. Willson, C.J. Kelly, and C.K. Glass, *The peroxisome proliferator-activated receptor-gamma is a negative regulator of macrophage activation.* Nature, 1998. **391**(6662): p. 79-82.

48. Wang, A.C., X. Dai, B. Luu, and D.J. Conrad, *Peroxisome proliferator-activated receptor-gamma regulates airway epithelial cell activation.* Am J Respir Cell Mol Biol, 2001. **24**(6): p. 688-93.

49. Reddy, R.C., V.R. Narala, V.G. Keshamouni, J.E. Milam, M.W. Newstead, and T.J. Standiford, *Sepsis-induced inhibition of neutrophil chemotaxis is mediated by activation of peroxisome proliferator-activated receptor-{gamma}.* Blood, 2008. **112**(10): p. 4250-8.

50. Birrell, M.A., H.J. Patel, K. McCluskie, S. Wong, T. Leonard, M.H. Yacoub, and M.G. Belvisi, *PPAR-gamma agonists as therapy for diseases involving airway neutrophilia.* Eur Respir J, 2004. **24**(1): p. 18-23.

51. Liu, D., B.X. Zeng, S.H. Zhang, Y.L. Wang, L. Zeng, Z.L. Geng, and S.F. Zhang, *Rosiglitazone, a peroxisome proliferator-activated receptor-gamma agonist, reduces acute lung injury in endotoxemic rats.* Crit Care Med, 2005. **33**(10): p. 2309-16.

52. Cuzzocrea, S., B. Pisano, L. Dugo, A. Ianaro, P. Maffia, N.S. Patel, R. Di Paola, A. Ialenti, T. Genovese, P.K. Chatterjee, M. Di Rosa, A.P. Caputi, and C. Thiemermann, *Rosiglitazone, a ligand of the peroxisome proliferator-activated receptor-gamma, reduces acute inflammation.* Eur J Pharmacol, 2004. **483**(1): p. 79-93.

53. Cuzzocrea, S., N.S. Wayman, E. Mazzon, L. Dugo, R. Di Paola, I. Serraino, D. Britti, P.K. Chatterjee, A.P. Caputi, and C. Thiemermann, *The cyclopentenone prostaglandin 15-deoxy-Delta(12,14)-prostaglandin J(2) attenuates the development of acute and chronic inflammation.* Mol Pharmacol, 2002. **61**(5): p. 997-1007.

54. Serhan, C.N., T. Takano, N. Chiang, K. Gronert, and C.B. Clish, *Formation of endogenous "antiinflammatory" lipid mediators by transcellular biosynthesis. Lipoxins and aspirin-triggered lipoxins inhibit neutrophil recruitment and vascular permeability.* Am J Respir Crit Care Med, 2000. **161**(2 Pt 2): p. S95-S101.

55. Gewirtz, A.T., B. McCormick, A.S. Neish, N.A. Petasis, K. Gronert, C.N. Serhan, and J.L. Madara, *Pathogen-induced chemokine secretion from model intestinal epithelium is inhibited by lipoxin A4 analogs.* J Clin Invest, 1998. **101**(9): p. 1860-9.

56. Sobrado, M., M.P. Pereira, I. Ballesteros, O. Hurtado, D. Fernandez-Lopez, J.M. Pradillo, J.R. Caso, J. Vivancos, F. Nombela, J. Serena, I. Lizasoain, and M.A. Moro, *Synthesis of lipoxin A4 by 5-lipoxygenase mediates PPARgamma-dependent, neuroprotective effects of rosiglitazone in experimental stroke.* J Neurosci, 2009. **29**(12): p. 3875-84.

57. Darley-Usmar, V.M., A. Hersey, and L.G. Garland, *A method for the comparative assessment of antioxidants as peroxyl radical scavengers.* Biochem Pharmacol, 1989. **38**(9): p. 1465-9.

58. Jame, A.J., P.M. Lackie, A.M. Cazaly, I. Sayers, J.F. Penrose, S.T. Holgate, and A.P. Sampson, *Human bronchial epithelial cells express an active and inducible biosynthetic pathway for leukotrienes B4 and C4.* Clin Exp Allergy, 2007. **37**(6): p. 880-92.

59. Bonfield, T.L., M.J. Thomassen, C.F. Farver, S. Abraham, M.T. Koloze, X. Zhang, D.M. Mosser, and D.A. Culver, *Peroxisome proliferator-activated receptor-gamma regulates the expression of alveolar macrophage macrophage colony-stimulating factor.* J Immunol, 2008. **181**(1): p. 235-42.

60. Lee, K.S., S.J. Park, P.H. Hwang, H.K. Yi, C.H. Song, O.H. Chai, J.S. Kim, M.K. Lee, and Y.C. Lee, *PPAR-gamma modulates allergic inflammation through up-regulation of PTEN.* FASEB J, 2005. **19**(8): p. 1033-5.

61. Birnbaum, Y., Y. Ye, Y. Lin, S.Y. Freeberg, S.P. Nishi, J.D. Martinez, M.H. Huang, B.F. Uretsky, and J.R. Perez-Polo, *Augmentation of myocardial production of 15-epi-lipoxin-a4 by pioglitazone and atorvastatin in the rat.* Circulation, 2006. **114**(9): p. 929-35.

62. Ye, Y., Y. Lin, S. Manickavasagam, J.R. Perez-Polo, B.C. Tieu, and Y. Birnbaum, *Pioglitazone protects the myocardium against ischemia-reperfusion injury in eNOS and iNOS knockout mice.* Am J Physiol Heart Circ Physiol, 2008. **295**(6): p. H2436-46.

63. Berger, W., M.T. De Chandt, and C.B. Cairns, *Zileuton: clinical implications of 5-Lipoxygenase inhibition in severe airway disease.* Int J Clin Pract, 2007. **61**(4): p. 663-76.

64. Collin, M., A. Rossi, S. Cuzzocrea, N.S. Patel, R. Di Paola, J. Hadley, M. Collino, L. Sautebin, and C. Thiemermann, *Reduction of the multiple organ injury and dysfunction caused by endotoxemia in 5-lipoxygenase knockout mice and by the 5-lipoxygenase inhibitor zileuton.* J Leukoc Biol, 2004. **76**(5): p. 961-70.

65. Ochalski, S.J., D.A. Hartman, M.T. Belfast, T.L. Walter, K.B. Glaser, and R.P. Carlson, *Inhibition of endotoxin-induced hypothermia and serum TNF-alpha levels in CD-1 mice by various pharmacological agents.* Agents Actions, 1993. **39 Spec No**: p. C52-4.

66. Hanefeld, M., N. Marx, A. Pfutzner, W. Baurecht, G. Lubben, E. Karagiannis, U. Stier, and T. Forst, *Anti-inflammatory effects of pioglitazone and/or simvastatin in high cardiovascular risk patients with elevated high sensitivity C-reactive protein: the PIOSTAT Study.* J Am Coll Cardiol, 2007. **49**(3): p. 290-7.

67. Pitocco, D., S. Giubilato, F. Zaccardi, E. Di Stasio, A. Buffon, L.M. Biasucci, G. Liuzzo, F. Crea, and G. Ghirlanda, *Pioglitazone reduces monocyte activation in type 2 diabetes.* Acta Diabetol, 2009. **46**(1): p. 75-7.

68. Schaller, G., J. Kolodjaschna, J. Pleiner, F. Mittermayer, S. Kapiotis, L. Schmetterer, and M. Wolzt, *Pioglitazone does not affect vascular or inflammatory responses after endotoxemia in humans.* Horm Metab Res, 2008. **40**(8): p. 549-55.

69. Boujoukos, A.J., G.D. Martich, E. Supinski, and A.F. Suffredini, *Compartmentalization of the acute cytokine response in humans after intravenous endotoxin administration.* J Appl Physiol, 1993. **74**(6): p. 3027-33.

70. Budde, K., H.H. Neumayer, L. Fritsche, W. Sulowicz, T. Stompor, and D. Eckland, *The pharmacokinetics of pioglitazone in patients with impaired renal function.* Br J Clin Pharmacol, 2003. **55**(4): p. 368-74.

71. Awni, W.M., R.A. Braeckman, G.R. Granneman, G. Witt, and L.M. Dube, *Pharmacokinetics and pharmacodynamics of zileuton after oral administration of single and multiple dose regimens of zileuton 600mg in healthy volunteers.* Clin Pharmacokinet, 1995. **29 Suppl 2**: p. 22-33.

72. Larsson, B.M., M. Kumlin, B.M. Sundblad, K. Larsson, S.E. Dahlen, and L. Palmberg, *Effects of 5-lipoxygenase inhibitor zileuton on airway responses to inhaled swine house dust in healthy subjects.* Respir Med, 2006. **100**(2): p. 226-37.

73. Dube, L.M., L.J. Swanson, and W. Awni, *Zileuton, a leukotriene synthesis inhibitor in the management of chronic asthma. Clinical pharmacokinetics and safety.* Clin Rev Allergy Immunol, 1999. **17**(1-2): p. 213-21.

74. Jaakkola, T., J.T. Backman, M. Neuvonen, and P.J. Neuvonen, *Effects of gemfibrozil, itraconazole, and their combination on the pharmacokinetics of pioglitazone.* Clin Pharmacol Ther, 2005. **77**(5): p. 404-14.

75. Gilman, M.D., A.J. Fischman, V. Krishnasetty, E.F. Halpern, and S.L. Aquino, *Optimal CT breathing protocol for combined thoracic PET/CT.* AJR Am J Roentgenol, 2006. **187**(5): p. 1357-60.

76. Goerres, G.W., E. Kamel, T.N. Heidelberg, M.R. Schwitter, C. Burger, and G.K. von Schulthess, *PET-CT image co-registration in the thorax: influence of respiration.* Eur J Nucl Med Mol Imaging, 2002. **29**(3): p. 351-60.

# APPENDIX
