# Supplementary material for: The peroxisome proliferator-activated receptor agonist pioglitazone and 5-lipoxygenase inhibitor zileuton have no effect on lung inflammation in healthy volunteers by positron emission tomography in a single-blind placebo-controlled cohort study
Source: PLoS One. 2018 Feb 7;13(2):e0191783. doi: 10.1371/journal.pone.0191783 (PMC5802889; doi:10.1371/journal.pone.0191783)
Supplement: S7 File — (RTF) [file pone.0191783.s008.rtf]

  

Human Research Protection Office			
		Barnes Jewish Hospital 
		St. Louis Children's Hospital
			Washington University
660 South Euclid Ave., Campus Box 8089, St. Louis, MO 63110 Phone: (314) 633-7400 FAX: (314) 367-3041

IRB ID #:	201101731

To:		Delphine Chen

From:		The Washington University in St. Louis Institutional Review Board,
WUSTL		DHHS Federalwide Assurance #FWA00002284
BJH		DHHS Federalwide Assurance #FWA00002281
SLCH		DHHS Federalwide Assurance #FWA00002282

Re:	Noninvasive quantification of the pulmonary anti-inflammatory effect of the glitazones.


Approval Date:	02/08/12


Next IRB Approval
Due Before:	03/14/12

Type of Application:	Type of Application Review: 	Approved for Populations:
	
 New Project		 Full Board: 	 Children
 Continuing Review	Meeting Date: 02/08/12	 Prisoners
 Modification		 Expedited	 Pregnant Women, Fetuses, Neonates
				 Exempt	 Wards of State
			 Facilitated	 Decisionally Impaired
				
				
					   			

Source of Support:	
	Doris Duke Charitable Foundation

MATERIALS APPROVED

Protocol:
Protocol Version:  		1.4
Protocol Date:     		12/12/2011

Amendment Number/Date(s):	1  -  04/26/2011
4  -  09/01/2011
5  -  09/29/2011
2  -  06/27/2011
3  -  07/26/2011 

Investigational Drug:
Investigational New Drug/Biologic Name:	E. coli O:113, Reference Endotoxin
Investigational New Drug/Biologic Number:	100042
Name of Sponsor who holds IND:	WUSTL Investigator (Delphine Chen, MD)
Investigator's Brochure Version(s):		Letter from Anthony Suffredini, MD at the NIH, dated January 4, 2006
Investigator's Brochure Dates(s):		Master File Number BB-MF 7294


Consent/Assent Materials:
	Consent & Assent Forms
		Form2_CONSENT_DDCF_IRB_rv20120128-1.rtf


Recruitment/Advertisement Materials:
	Recruitment: Advertisements
		Chen 201101731_website_rv_9-1-11.rtf
		Chen_201101731_flyer_rv_9-1-11.rtf
		Chen 201101731 - BJC Today_rv_.rtf
		Chen_201101731_Centerwatch_rv_9-1-11.rtf
		Chen_201101731_Facebook_rv_9-1-11.rtf


Questionnaires:
	Subject Data Collection Instruments
		Study_Letter_DDCF_rev20111212.rtf
		Phone Call Log 201101731.docx
		Med Diary 201101731 rev20111212.docx
		PreScreening_Form_DDCF_rev20111212.rtf
		Telephone_Script_DDCF_20111212.rtf


This approval has been electronically signed by IRB Chair or Chair Designee:
Carissa Minder, BSN, RN
02/09/12 1503
IRB Approval:  IRB approval indicates that this project meets the regulatory requirements for the protection of human subjects.  IRB approval does not absolve the principal investigator from complying with other institutional, collegiate, or departmental policies or procedures.

Recruitment/Consent:  Your IRB application has been approved for recruitment of subjects not to exceed the number indicated on your application form.  If you are using written informed consent, the IRB-approved and stamped Informed Consent Document(s) are available in myIRB.  The original signed Informed Consent Document should be placed in your research files.  A copy of the Informed Consent Document should be given to the subject.  (A copy of the signed Informed Consent Document should be given to the subject if your Consent contains a HIPAA authorization section.)  

Continuing Review:  Federal regulations require that the IRB re-approve research projects at intervals appropriate to the degree of risk, but no less than once per year.  This process is called “continuing review.”  Continuing review for non-exempt research is required to occur as long as the research remains active for long-term follow-up of research subjects, even when the research is permanently closed to enrollment of new subjects and all subjects have completed all research-related interventions and to occur when the remaining research activities are limited to collection of private identifiable information. Your project “expires” at midnight on the date indicated on the preceding page (“Next IRB Approval Due on or Before”).  You must obtain your next IRB approval of this project by that expiration date.  You are responsible for submitting a Continuing Review application in sufficient time for approval before the expiration date, however you will receive reminder notice prior to the expiration date.

Modifications:  Any change in this research project or materials must be submitted on a Modification application to the IRB for prior review and approval, except when a change is necessary to eliminate apparent immediate hazards to subjects.  The investigator is required to promptly notify the IRB of any changes made without IRB approval to eliminate apparent immediate hazards to subjects using the Modification/Update Form. Modifications requiring the prior review and approval of the IRB include but are not limited to:  changing the protocol or study procedures, changing investigators or funding sources, changing the Informed Consent Document, increasing the anticipated total number of subjects from what was originally approved, or adding any new materials (e.g., letters to subjects, ads, questionnaires).

Unanticipated Problems Involving Risks:  You must promptly report to the IRB any unexpected adverse experience, as defined in the IRB/HRPO policies and procedures, and any other unanticipated problems involving risks to subjects or others.  The Reportable Events Form (REF) should be used for reporting to the IRB.

Audits/Record-Keeping:  Your research records may be audited at any time during or after the implementation of your project.  Federal and University policies require that all research records be maintained for a period of seven (7) years following the close of the research project.  For research that involves drugs or devices seeking FDA approval, the research records must be kept for a period of three years after the FDA has taken final action on the marketing application, if that is longer than seven years.

Additional Information:  Complete information regarding research involving human subjects at Washington University is available in the “Washington University Institutional Review Board Policies and Procedures.”  Research investigators are expected to comply with these policies and procedures, and to be familiar with the University's Federalwide Assurance, the Belmont Report, 45CFR46, and other applicable regulations prior to conducting the research.  This document and other important information is available on the HRPO website http://hrpohome.wustl.edu/. 
